# Machine learning-accelerated quantum mechanics-based atomistic simulations for industrial applications

**DOI:** 10.1007/s10822-020-00346-6

**Published:** 2020-10-09

**Authors:** Tobias Morawietz, Nongnuch Artrith

**Affiliations:** 1 Bayer AG, Pharmaceuticals, R&D, Digital Technologies, Computational Molecular Design, 42096 Wuppertal, Germany; 2 Department of Chemical Engineering, Columbia University, New York, NY 10027 USA

**Keywords:** Quantum mechanics, Machine learning, Neural networks, Drug discovery, Energy materials, Industrial applications

## Abstract

Atomistic simulations have become an invaluable tool for industrial applications ranging from the optimization of protein-ligand interactions for drug discovery to the design of new materials for energy applications. Here we review recent advances in the use of machine learning (ML) methods for accelerated simulations based on a quantum mechanical (QM) description of the system. We show how recent progress in ML methods has dramatically extended the applicability range of conventional QM-based simulations, allowing to calculate industrially relevant properties with enhanced accuracy, at reduced computational cost, and for length and time scales that would have otherwise not been accessible. We illustrate the benefits of ML-accelerated atomistic simulations for industrial R&D processes by showcasing relevant applications from two very different areas, drug discovery (pharmaceuticals) and energy materials. Writing from the perspective of both a molecular and a materials modeling scientist, this review aims to provide a unified picture of the impact of ML-accelerated atomistic simulations on the pharmaceutical, chemical, and materials industries and gives an outlook on the exciting opportunities that could emerge in the future.

## Introduction

Computational methods play an increasingly important role in R&D processes across the pharmaceutical, chemical, and materials industries. Computer-aided drug design [1–3] has the potential to lower the cost, decrease the failure rates, and speed up the discovery process. Computational materials methods help to identify novel materials [4, 5], for example, for renewable energy applications [6] such as catalytic energy conversion [7] and energy storage [8]. Results from atomistic simulations aid in the interpretation of experimental measurements and give insights into the structure, dynamics and mechanisms of processes occurring on the atomic scale.

In the last decades a new class of atomistic simulation techniques has emerged that combines machine learning (ML) with simulation methods based on quantum mechanical (QM) calculations. Such ML-based acceleration can dramatically increase the computational efficiency of QM-based simulations and enable to reach the large system sizes and long timescales required to access properties with relevance for industry.

Here, we review a selection of ML-accelerated QM methods and their applications to drug design and materials discovery. In the next section we briefly summarize the two main conventional approaches for atomistic simulations, based on molecular mechanics (MM) and QM, respectively, and we show how ML can help overcome their limitations. This is followed by a discussion of recent methodological advances in ML-based interatomic potentials (force fields) for the modeling of complex molecular and materials systems. Finally, we review recent applications of these methods in the fields of drug discovery and materials design. We show that ML-accelerated QM simulations have now matured to the point where they can have a large impact on industrial processes.

## Atomistic simulation methods

**Fig. 1:**
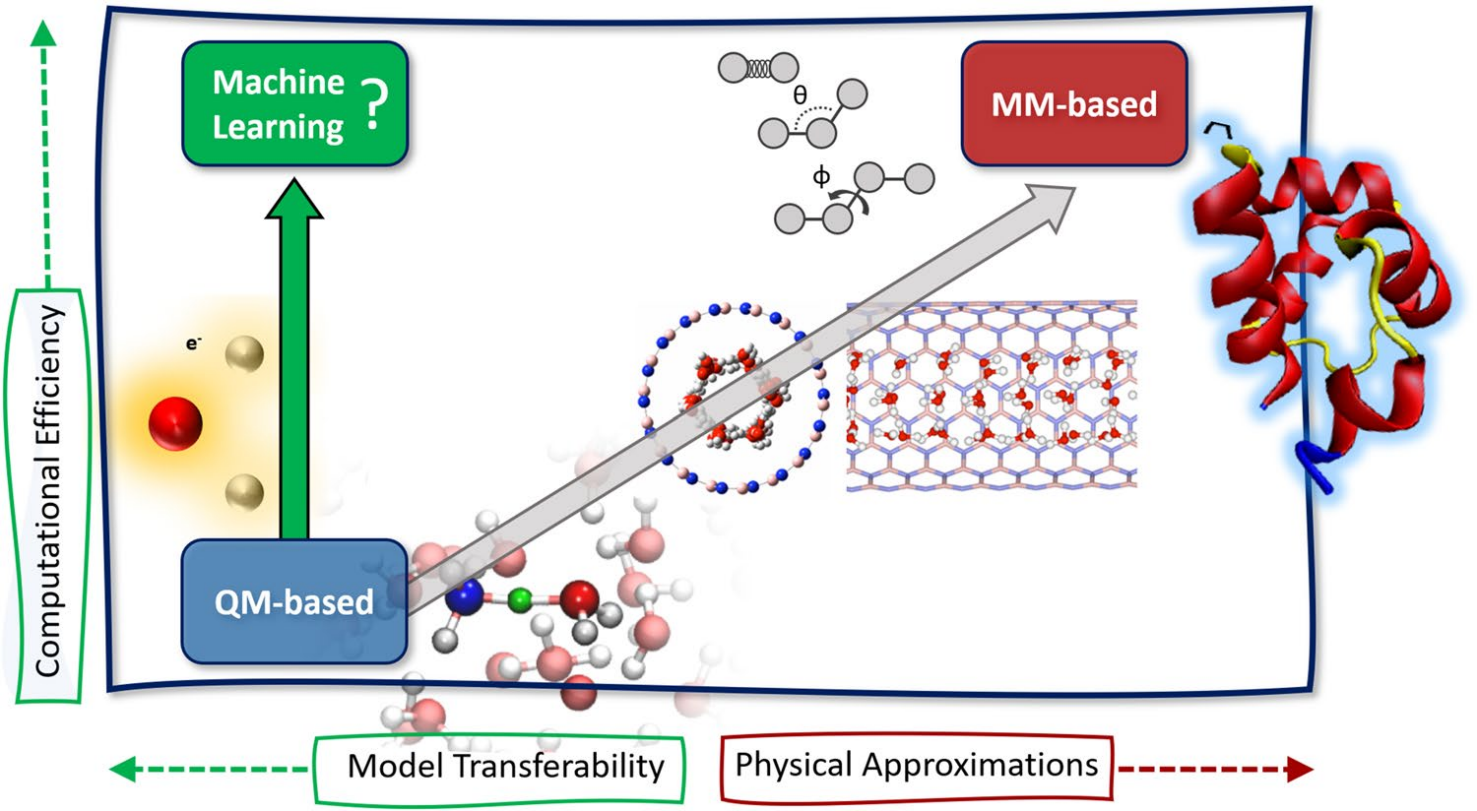
Atomistic simulation methods can be broadly categorized into two classes depending on the way the system is described: using quantum mechanical (QM) calculations based on the electronic structure or molecular mechanics (MM) methods with predefined functional forms. Due to their higher computational cost QM-based simulations are limited to smaller systems while MM-based methods are more efficient but rely on many approximations and are often derived from experimental input. The goal of QM-based machine learning is to raise the efficiency of QM methods without sacrificing their transferability, predictive power and ability to describe complex bonding patterns including the breaking and forming of chemical bonds

The central quantity in atomistic simulations is the potential-energy surface (PES), a high-dimensional function of the position of all atoms in the system. The potential energy is the basic ingredient for Monte Carlo (MD) simulations, while the derivative of the PES yields the atomic forces that are required to numerically solve Newton’s equations of motion in molecular dynamics (MD) simulations [9, 10]. The choice between MD and MC simulations depends on the system and physical process one wants to investigate. MC methods can be employed to obtain structural properties, are efficient in overcoming energy barriers, and can be used for simulating processes in which the number of particles varies. The continuous trajectories generated in MD simulations on the other hand allow to obtain dynamic properties such as vibrational spectra and diffusion coefficients.

When applying atomistic simulations to a given research question, one of the most important considerations is the choice of the simulation method that describes the PES and produces the energy and (possibly) atomic forces that drive the simulation. Depending on the degree of *physical approximation*, simulation methods are more or less *computationally efficient*.

Physically most accurate and computationally most expensive are non-empirical QM-based methods that describe the electronic structure and the atomic structures with all degrees of freedom. QM-based simulations are typically limited to small system sizes of less than thousand atoms and short time scales on the order of picoseconds. On the other end of the scale are simulation methods based on empirical molecular mechanics (MM) that do not explicitly describe the electronic structure and may additionally coarse-grain atomic structures by removing select degrees of freedom. Simulation methods that are direct non-empirical approximations to QM are (usually) *transferable* across the periodic table and across different atomic structures (e.g., organic molecules, bio-polymers, inorganic solids), whereas empirical methods are parametrized for a specific application and are typically not transferable to other situations.

Finally, the *usability* of a simulation method also depends on the availability of accessible and well-documented software implementations. Hence, the choice of simulation method depends on the physical approximation that is called for by the given research question, and is generally informed by the following four aspects:

1. The types of *physical approximations* made,
2. The *computational efficiency* of the method,
3. Its *transferability*, and
4. Its *usability*.

Note that a specific research question also determines the relevant length and time scales (e.g., proteins vs. small molecules), and a given application might simultaneously call for high physical accuracy and large length/long time scales. Such research questions cannot be addressed with conventional simulation methods. Novel ML methods, discussed in Sect. Machine learning potentials for atomistic simulations, can overcome this limitation.

A schematic overview of the interrelationship of physical approximation and computational efficiency is shown in Fig. 1. In the following, we briefly review conventional atomistic simulation methods before discussing how these methods can be accelerated and generalized using ML techniques.

### MM-based simulations

In MM-based simulations analytical functions with a small number of parameters often derived from experimental input are employed to describe the PES. They are typically developed for a specific system or application and are called *force fields* in the context of bio-molecular simulations [11] or *interatomic potentials* for the description of materials systems [12]. Commonly used force fields (such as AMBER [13], CHARMM [14], GROMOS [15], and OPLS [16]) are computationally very efficient since they employ simple pairwise interaction terms and fixed atomic charges. They also rely on the definition of atomic connectivities and atom types and are therefore non-reactive. Examples for interatomic potentials for the descriptions of solids and surfaces are the Lennard-Jones pair potential [17], the embedded atom model (EAM) [18], and bond order potentials such as the Tersoff potential [19].

MD simulations with force fields have become a key technique for different stages in the drug discovery pipeline [20, 21]. One specific example is their use in the early discovery phase for the calculation of relative binding affinities of ligand molecules to a protein binding site. The ability to efficiently calculate the associated binding free energy [22–24] can provide valuable contributions to the ligand optimization phase, allowing to rank ligands, optimize selectivity, and estimate off-target interactions. With recent advances in the free energy methods and underlying simulation models, the calculation of free energies from atomistic MD simulations has become a reliable tool with several examples of successful industrial applications [25–29]. Multi-scale approaches in which atomistic force fields are combined with coarse-grained models that were built for specific applications can further reduce the computational cost and allow to study even larger systems such as membrane-bound ion channels [30].

A general drawback of empirical MM simulations is the fact that the obtained results depend on the experimental data the models are based on, which restricts their predictive power and transferability to conditions not included in the optimization process. Standard force fields for example are parametrized to a limited set of chemical elements and cannot be easily applied to metal-containing proteins. Another serious limitation is the inability to describe the breaking and forming of chemical bonds, prohibiting their use for industrially relevant processes such as the investigation of enzymatic reactions for covalent inhibitor design [31] or of catalytic reactions at metal oxide surfaces [32].

To address these limitations several extensions of empirical force fields and potentials have been developed. Examples are approaches that go beyond atom types using the SMARTS chemical perception language [33] developed by the Open Force Field Initiative [34], reactive force fields that allow the breaking of chemical bonds [35], and the development of frameworks for systematic and reproducible parametrization procedures [36, 37]. All these approaches have in common that the employed functional form to approximate the PES is predetermined and for the sake of efficiency approximated by simplified functions with a small number of model parameters.

### QM-based simulations

QM-based simulation methods (also called *ab initio* molecular dynamics [AIMD] or *first principles* simulations) [38, 39] circumvent the problem of defining a functional form for the PES. Here, energy and atomic forces are obtained *on-the-fly*, in each step of the simulation, by (approximately) solving the Schrödinger equation using an electronic structure method like density-functional theory (DFT) [40]. QM simulations are fully reactive and can describe the complex bonding patterns, polarization effects and charge transfer processes that govern the behaviour of biological systems [41]. In combination with path integral approaches also nuclear quantum effects (NQEs) [42, 43] like zero-point motion and tunneling can be included, processes that are important for the description of systems containing hydrogen-bond networks and acidic protons. QM-based simulations can be applied to obtain a large set of materials properties, for example the stability of crystal structures, elastic constants, and transport phenomena. In addition to energies and forces, other observables can be directly calculated by QM methods such as dipole moments, polarizabilities, chemical shifts, and phonon frequencies for the spectroscopic characterization of molecular and materials systems.

While MM-based simulations are only possible when reliable force fields (or interatomic potentials) for the given system are available, QM methods are in principle applicable to all chemical species. In practice, there is no single QM method that is computationally affordable and reliable for every system, and the approximations made in a chosen method still need to be carefully validated [40]. The largest bottleneck of QM-based simulations is the high computational cost of the electronic structure calculations that have to be executed in each simulation step. Even for efficient QM methods such as DFT the algorithmic scaling is typically of order ${\mathcal {O}}(N^3)$ in the number of electrons *N*, which means that an increase in system size by a factor of 10 leads to an increase in processing time of a factor of 1000. This severely limits the application of QM simulations to small, often idealized model structures containing not more than a few hundred atoms. Semi-empirical methods [44–47] such as density functional tight binding (DFTB) [48–50] lower the computational burden and can even describe full proteins [51] but their efficiency comes at the cost of transferability and accuracy.

### Overcoming the limitations of QM-based simulations with machine learning

As detailed in the previous two sections, MM-based atomistic simulation methods can be computationally *highly efficient* but have limited transferability owing to their high degree of physical approximation. Conversely, QM-based methods can be *highly accurate* but are computationally too demanding for many applications of industrial relevance. While mixed quantum mechanics/molecular mechanics (QM/MM) approaches [52–55] can, in principle, combine the strengths of both worlds, QM/MM is technically involved and usability is therefore not always given. Modifications [56, 57] of the original Car-Parrinello method [38] can reduce the computational burden of QM simulations to some extend but are still much more costly compared to MM-based simulations. If a research question requires simultaneously high accuracy and high computational efficiency (the top left corner of the schematic in Fig. 1), this means in practice often that it cannot be addressed with conventional atomistic simulation methods.

To overcome this limitation, a number of methodologies based on ML have been developed during the last decades. The purpose of the different ML strategies generally falls into one of the following four categories:

1. Extension of the applicability range of QM simulations to larger length and time scales;
2. Prediction of properties calculated from QM methods;
3. Automated analysis of simulation data; and
4. Inversion of atomistic calculations to generate atomic structures for a given set of properties.

Strategy (1) is based on the development of machine-learning potentials (MLP) that achieve an accuracy that is close to (or identical to) QM-based methods but at significantly reduced computational cost that scales linearly with the system size. MLPs can be taken as drop-in replacement for conventional interatomic potentials or force fields, which ensures a high usability.

In strategy (2), ML models are trained to yield the outcome of QM-based calculations either using optimized structures or configurations obtained from atomistic simulations. Examples are ML predictions of atomization energies [58, 59] of small organic molecules, nuclear magnetic resonance (NMR) shifts [60] and band gaps [61–63] of inorganic solids, and adsorption energies of electrocatalysts [64]. By design, ML models of type (2) are less general than MLPs as they are specific to one or few QM properties and do not easily transfer to others. The increasing availability of QM databases enables training such ML models for an ever growing number of QM properties, and we discuss examples in Sect. Spectroscopic techniques for structure characterization.

MD and MC simulations of complex atomistic systems can yield data that are challenging or time-consuming to interpret for humans, such as MD trajectories with frames (atomic coordinates) from billions of time steps. Strategy (3) uses ML techniques for the analysis of simulation data, for example for the automatic identification of crystal structures [65] or the extraction of free energy surfaces from enhanced-sampling MD simulations [66, 67].

Finally, the *inverse design* strategy (4) holds great promise for the future of molecular and materials design but is currently in its infancy with few published examples. For examples of inverse molecular design, we refer to a recent review by Sanchez-Lengelin and Aspuru-Guzik [68]. In general, methods that implement ML models of type (4) are not yet standardized and usability is therefore generally not yet given.

In this review we focus on ML approaches of strategy (1) and (2), i.e. MLPs for accelerated simulations and ML models that predict the outcome of QM calculations, since those are the most mature and offer a reasonable balance of usability and pay-off for industrial applications. More general applications of ML approaches, for example for retrosynthesis [69, 70], direct prediction of experimental properties [71–73], and molecule generation and optimization [74–76], are discussed in references [77–80]. In the following section, we discuss different types of MLPs and approaches for their construction.

**Fig. 2:**
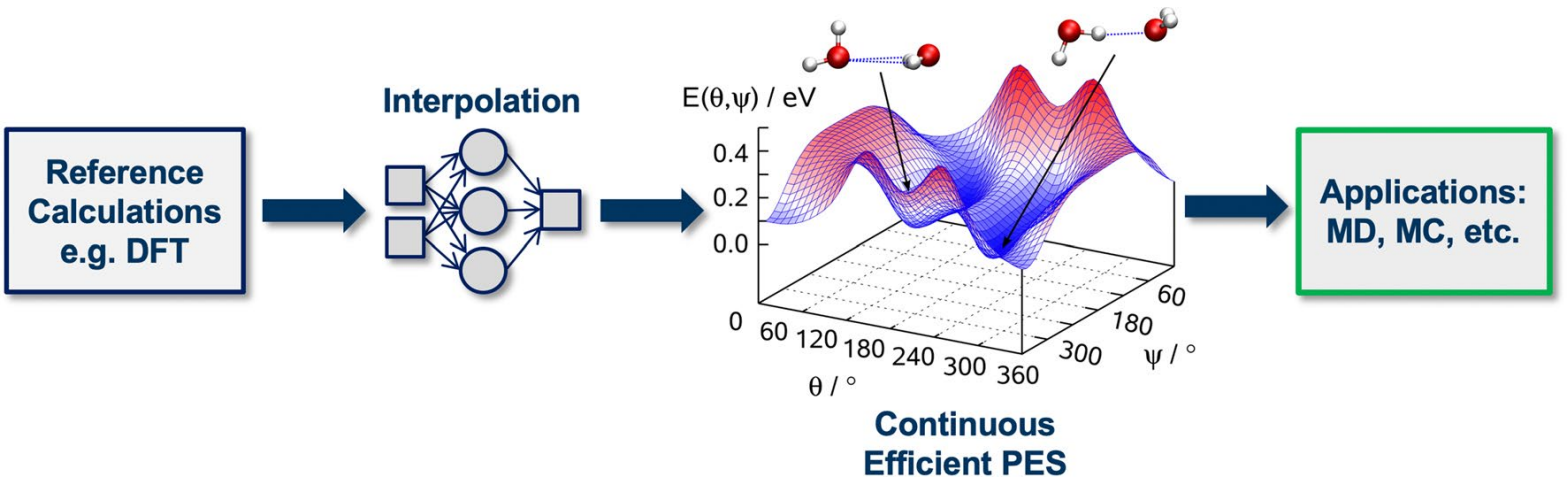
Workflow for machine learning-accelerated atomistic simulations: first, reference calculations are performed for a set of configurations using a quantum mechanical (QM) method such as density-functional theory (DFT). The resulting QM energies (and potentially forces) are then used to train a machine learning model that maps the atomic structure to its corresponding energy and by that learns the potential-energy surface (PES) of the atomistic system. Once trained, the resulting ML model yields a continuous representation of the PES that can be efficiently evaluated and allows to perform molecular dynamics (MD) or Monte Carlo (MD) simulations for larger systems and on longer time-scales than possible with direct QM-based simulations

## Machine learning potentials for atomistic simulations

As discussed in the previous section, atomistic simulations sample the PES of collections of atoms, and the description of the PES may be either based on first principles QM or on approximate physical or *ad-hoc* mathematical expressions. If a PES is described by a mathematical function that does not have any direct correspondence in the laws of physics, the PES can nevertheless be highly accurate if it interpolates the true QM potential energy faithfully for all relevant atomic arrangements. For example, the mathematical form of the repulsive branch of the (12-6) Lennard-Jones pair potential [17] was originally chosen for computational simplicity and does not reflect the true exponential behavior known from QM, but the Lennard-Jones potential nevertheless describes noble gas dimers with great accuracy because it provides a good interpolation of the QM potential energy for all relevant bond lengths. An atomistic simulation of an argon dimer with a Lennard-Jones potential can therefore be just as accurate as a full quantum-mechanical calculation, while it is computationally more efficient by several orders of magnitude.

*What if we had a flexible mathematical function that is able to accurately interpolate the QM potential energy for any arbitrary atomic system, not only for dimers or select classes of materials?*

As it turns out, it can be shown that artificial neural networks (ANN) [81] with finite numbers of parameters can represent any real-valued continuous function, such as PESs, with arbitrary accuracy. This is in simple terms the conclusion of the *universal approximation theorem*[82, 83], and similar theorems have been derived also for other ML regression methods such as Gaussian process regression (GPR) [84]. Hence, carefully constructed ML regression models can in principle replace any QM PES without loss of accuracy.

The regression or interpolation of PESs with ML, is at the core of *ML potentials*. Fig. 2 shows an overview of the main steps involved in the construction and application of ML potentials for accelerated QM-based simulations: *(1)* reference calculations, *(2)* model training, *(3)* model application. The various ML potential methods differ in the ML method used for regression and the *descriptor* approach used for the translation of atomic structures to *features* that are suitable as input for ML models.

Several ML methods have been used for the task of learning PESs, from ANNs [85, 86], to GPR [87, 88], and kernel ridge regression (KRR) [89]. The discussion here focuses on ANN-based ML potentials, which have been applied to the widest range of materials and compositions.

### Representation of PESs with ANNs

On a fundamental level, ANNs are non-linear vector functions that take a vector as input and produce another vector as output. The functional form of ANNs consists of a combination of elemental building blocks that may be interpreted as *artificial neurons*, since they perform an operation that is on a basic level similar to that of a biological neuron. Each artificial neuron takes the weighted sum of one or more input values $x_{i}$ and applies a non-linear *activation function* $f_{\text {a}}$ to the result

1 $$y = {f_{\text {a}}}{\left(\sum_{i} {{a_{i}} {x_{i}}} + b \right)}$$

where $a_{i}$ is the weight of the *i*-th input and *b* is a *bias* weight that allows for an additional constant shift that does not depend on the input values. An ANN is the combination of interconnected artificial neurons such that the outputs of some neurons are the inputs of others. In a *feed-forward ANN*, the neurons are organized in layers, and all connections are in one direction, i.e., outputs from all neurons of one layer are the inputs of the neuron of the subsequent layer. The graph representation of a feed-forward ANN is shown under the label “interpolation” in Fig. 2. *ANN training* is the process of optimizing the weight parameters $\{a_{i}\}$ and $\{b\}$ for each neuron to reproduce reference data within a *training set*.

In principle, feed-forward ANNs can be directly used for the interpolation of PESs in the sense that is indicated in Fig. 2. In this scheme, the atomic positions, e.g., the Cartesian coordinates of all atoms, are the input of the ANN, and the potential energy is the output. Variations of this approach have been used in theoretical chemistry since the 1990s [90–93] to accelerate the modeling of select molecular systems.

This naïve interpolation, however, disregards fundamental symmetries of the potential energy with respect to rotation/translation of the atomic structure and the exchange of equivalent atoms. Hence, care must be taken that the ANN-interpolated PES does not exhibit unphysical features. In addition, the input dimension of the ANN is fixed to the number of degrees of freedom of a specific atomistic system, and it is not possible to use the same ANN to predict the potential energy of atomic structures with fewer or more atoms.

Lorenz et al. [94] introduced a transformation of the Cartesian atomic coordinates into a set of coordinates that incorporates the symmetries of the PES before the ANN interpolation, to describe the dissociation of an ${{H}_2}$ molecule over the Pd(100) surface. The limitation to a fixed number of atoms was removed by the high-dimensional neural network potential approach.

### High-dimensional neural network potentials

To overcome the limitations of ANN-interpolated PESs, in 2007 Behler and Parrinello proposed an ANN potential methodology [85] that is based on an implicit decomposition of the total potential energy $E(\sigma )$ of an atomic structure $\sigma$ into atomic energy contributions $E_{i}$

2 $$E(\sigma) \approx \sum_{i}^{\text{atoms}} E_{i}(\sigma_{i}) {\text{with}} \quad \sigma_{i}= \left\{{\mathbf{R}}_{j}, t_{j} \; {\text{for}}\; | {\mathbf{R}}_{j} - {\mathbf{R}}_{i}|\le R_{\text{cut}}\right\}.$$

In equation (2), $\sigma _{i}$ is the *local structural environment* of atom *i* that contains only the coordinates ${\mathbf {R_{j}}}$ and chemical species $t_{j}$ of those atoms that are within a cutoff distance $R_{\text {cut}}$ from the position ${\mathbf {R}}_{i}$ of atom *i*. In the high-dimensional neural network potential method, ANNs are trained to predict the atomic energy $E_{i}$.

Following the idea by Lorenz et al. [94], the ANN input is obtained by representing features of the local atomic environment $\sigma _{i}$ with *symmetry functions* [85, 95] that incorporate the rotational symmetry and the symmetry with respect to the exchange of equivalent atoms. Other symmetry-invariant *descriptors* or *fingerprints* of local atomic environments have since been developed, and key methods are reviewed in Sect. Descriptor of the local atomic environment.

The original Behler-Parrinello method was limited to a single atomic species. In 2011, the method was extended to multicomponent compositions by Artrith, Morawietz, and Behler by training separate ANNs for different atomic types *t* [96]. The total energy in the multicomponent ANN potential method is given by

3 $$E(\sigma ) \approx E_{\textsc {ann}}(\sigma ) = \sum _{t}^{\begin{array}{c} \text {atom}\\ \text {types} \end{array}} \sum _{i}^{\begin{array}{c} \text {atoms of}\\ \text {type { t}} \end{array}} \text {ANN}_{t}(\widetilde{\sigma }_{i}),$$

where $\widetilde{\sigma }_{i}$ is the symmetry-invariant descriptor (fingerprint) of the local atomic environment $\sigma _{i}$ and $\text {ANN}_{t}$ is the atomic ANN for atoms of type *t*.

**Fig. 3:**
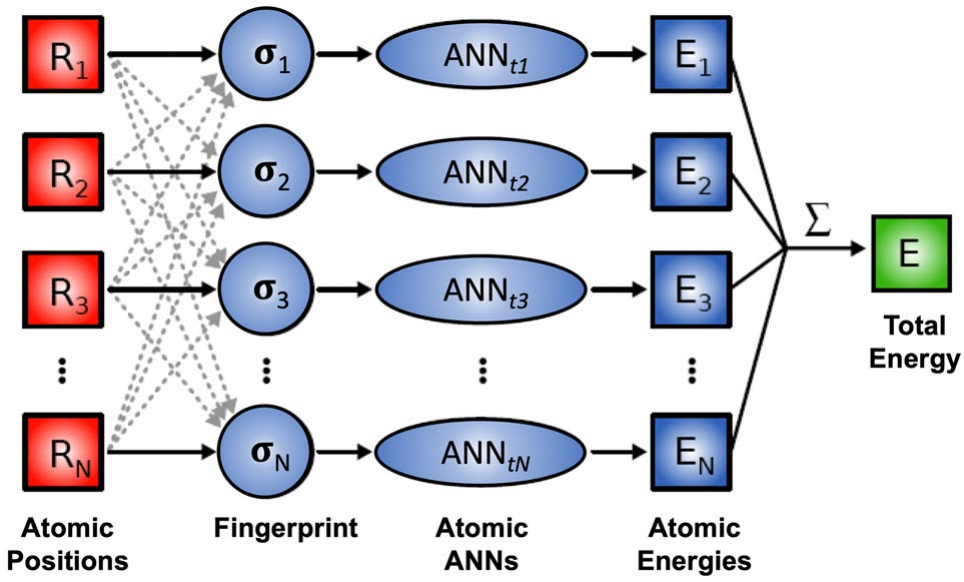
Diagram of the high-dimensional neural network that combines the atomic ANNs of all atoms in a structure for an *N*-atom system. The output is the total energy *E*, which is the sum of the individual atomic energy contributions $E_i$, which are in turn the outputs of atomic feed-forward ANNs

Figure 3 shows a graph representation of a high-dimensional neural network potential including the translation of the atomic coordinates ${\mathbf {R}}_{i}$ to an invariant fingerprint of the local atomic environment $\widetilde{\sigma }_{i}$ and the prediction of the atomic energies $E_{i}$ by the atomic ANNs.

Note that training ANN potentials that are based on atomic energy contributions is technically more involved than the direct ANN interpolation of the PESs discussed in Sect. Representation of PESs with ANNs, since the atomic energies are not uniquely defined in QM simulation methods and are therefore not directly available as reference. In most ANN potential methods the atomic energy is learned implicitly from the total energy, i.e., the reference data for the ANN potential training are total energies (and its derivatives). Alternatively, the QM total energies can be first decomposed into atomic energy contributions via non-unique schemes [97], which can improve the training efficiency but introduces an additional step in data preparation.

A fundamental assumption of the multicomponent ANN potential approach as expressed in Eq. (3) is that the total energy is entirely given by a sum of short-ranged atomic energy contributions. However, some contributions to the total energy are known to be long-ranged. Specifically, atomic structures with ionic species or ionic bonding contributions exhibit long-ranged electrostatic interactions. Also, long-ranged dispersive (*London* or *van der Waals*) interactions are of crucial importance, for example, for (bio)polymers and for adsorption phenomena.

### ANN potentials with long-ranged electrostatic interactions

The energy contribution from the electrostatic interaction of two charged atoms *i* and *j* is given by *Coulomb’s law*

4 $$E^{\text {elec}}_{i,j}({\mathbf {R}}_{i}, {\mathbf {R_{j}}}, q_{i}, q_{j}) = \frac{1}{4\pi \varepsilon _{0}} \frac{q_{i} q_{j}}{R_{ij}},$$

where $q_{i}$ and $q_{j}$ are the atomic charges, $R_{ij}=|{\mathbf {R}}_{j} - {\mathbf {R}}_{i}|$ is the interatomic distance, and $\varepsilon _{0}$ is the permittivity of the vacuum. Since Coulomb interactions decay only as $1/R_{ij}$ with the interatomic distance, they cannot be generally truncated at any cutoff, hence, the ANN potential expression of Eq. (3) would be inappropriate irrespective of the cutoff chosen for the local atomic environment. Note that electrostatic interactions in dense media, such as solids or liquids, are screened and can often be treated as effectively short-ranged [98]. It should be also kept in mind that the distance dependence of the ANN forces is twice as large as the chosen cutoff that defines the size of the local atomic environments [96, 99–101]. If screening cannot be assumed, the ANN potential approach needs to be extended.

**Fig. 4:**
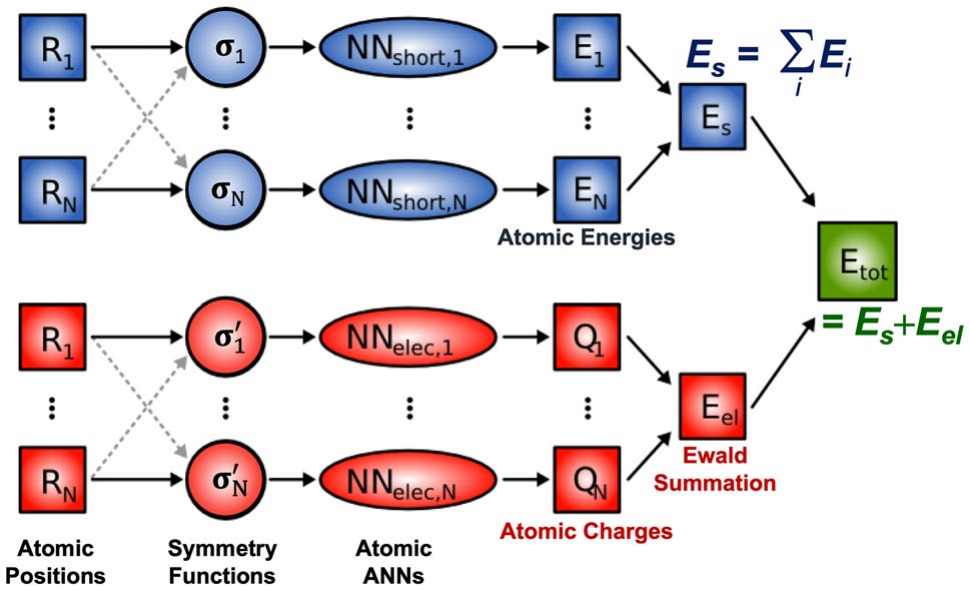
Diagram of the high-dimensional neural network potential for multicomponent systems: The total energy of the system is obtained as a sum of a short-range energy (E$_\text {s}$) obtained as shown in Fig. 3 and a long-range electrostatic energy (E$_\text {el}$), which is calculated from atomic charges $Q_i$. Both the short-range atomic energies and the atomic charges depend on the local atomic environments and are constructed by atomic ANNs [96]

To address this need, Artrith, Morawietz, and Behler proposed an extension of the ANN potential method by a second set of ANNs that are trained to reproduce environment-dependent atomic charges [96, 101–103]. In this approach, the total energy is given by

5 $$E_{\textsc {ann}}^{\text {total}} = E_{\textsc {ann}}^{\text {short}} + E_{\textsc {ann}}^{\text {long}},$$

where the short-range energy contribution $E_{\textsc {ann}}^{\text {short}}$ is given by the expression of Eq. (3). The long-range energy contribution takes the usual Coulomb form

6 $$E_{\textsc {ann}}^{\text {long}} = \sum _{i,j}^{\text {atoms}} E^{\text {elec}}_{i,j}({\mathbf {R}}_{i}, {\mathbf {R_{j}}}, q_{i}, q_{j}),$$

which can be evaluated, for example, with the Ewald summation technique [104] or using approximate damped techniques such as the pairwise approach by Fennell and Gezelter [105]. The atomic charges in Eq. (6), $q_{k}=\text {ANN}_{t}^{q}(\widetilde{\sigma }_{k})$, are represented by ANNs as function of the local atomic environment. A schematic of the electrostatic extension of the high-dimensional ANN potential method is shown in Fig. 4.

The original approach [96, 102] trained the charge ANNs on Hirshfeld charges [106]. Since the decomposition of the total charge density into atomic contributions is not uniquely defined, other charge partitioning schemes [107] would have been equally valid. To avoid training potentially ill-defined atomic charges directly, Yao et al. trained atomic charges implicitly such that they reproduce molecular dipole moments [108], which are physical observables. In the case of ionic crystals, a static charge approach in which the atomic charges are independent of the environment has also been demonstrated to work [109]. Finally, the restriction of long-ranged electrostatic interactions to the Coulomb form and to explicit atomic charges might be avoidable by introducing an energy term that depends on long-ranged features of the atomic structure, which Grisafi and Ceriotti recently demonstrated for a simplified model [110].

### ANN potentials with dispersive interactions

In addition to splitting off electrostatic interactions, also dispersive van der Waals (vdW) interactions can be treated separately. Morawietz and Behler [111] introduced an extended energy expression,

7 $$E^{\text {total}} = E_{\textsc {ann}}^{\text {short}} + E_{\textsc {ann}}^{\text {elec}} + E^{\text {disp}},$$

where $E^{\text {disp}}$ is an analytic correction term which improves the description of dispersion interactions using Grimme’s D3 method [112]. A similar approach was taken by Yao et al. [108] based on the D2 correction scheme [113].

While the main reason for introducing explicit electrostatics is their long-ranged nature which cannot be represented by short-range atomic energies, the need to include a separate dispersion term depends on the employed reference method and the system. A vdW correction term might be required if DFT is used as the reference method, since (semi-)local density-functionals suffer from an inaccurate description of these interactions. Having an explicit vdW correction term that is added to the short-range energy represented by the ANN has the additional benefit that its interactions are not truncated at a short distance. However, for the description of homogeneous systems in which long-range forces are screened, it is also valid to add the vdW term to the short-range reference data and train ANNs on the joined energies and forces. This was for example done to study the impact of vdW interactions on the properties of ice and liquid water by training ANNs to represent two density-functionals with and without inclusion of a vdW correction term [114].

### Descriptor of the local atomic environment

The explicit or implicit decomposition of the total structural energy, either into atomic contributions as in Eq. (2) or into the contributions of bonds or other fragments is a general feature of transferable ANN potentials. However, the various ANN potential methods developed today differ often in the symmetry-invariant descriptor (fingerprint) used for the feature extraction from atomic or fragment environments.

Widely adopted descriptor methods are based on the expansion of the atomic positions or bond-length and angle distributions. Recently, Xie and Grossman proposed a graph convolution approach as descriptor for molecular and periodic atomic structures [115]. This descriptor was further adapted by Chen et al. [116], who applied it to the development of accurate ML models for property prediction. While most ANN potential methods rely on *hand-crafted* descriptors that were designed based on chemical intuition, the recent deep ANN potential method by Schütt et al. [117] avoids the need for empirical feature extraction by means of a general convolution approach akin to those used in computer vision.

The choice of the optimal descriptor method depends on the application, as some methods are better suited for isolated molecular systems where others were designed for periodic solids. Another factor in the descriptor selection is the balance of computational efficiency and accuracy. Various approaches have been proposed [88, 95, 118–126], and we limit the discussion here to the ones that are most commonly used and are available in public software implementations.

**Fig. 5:**
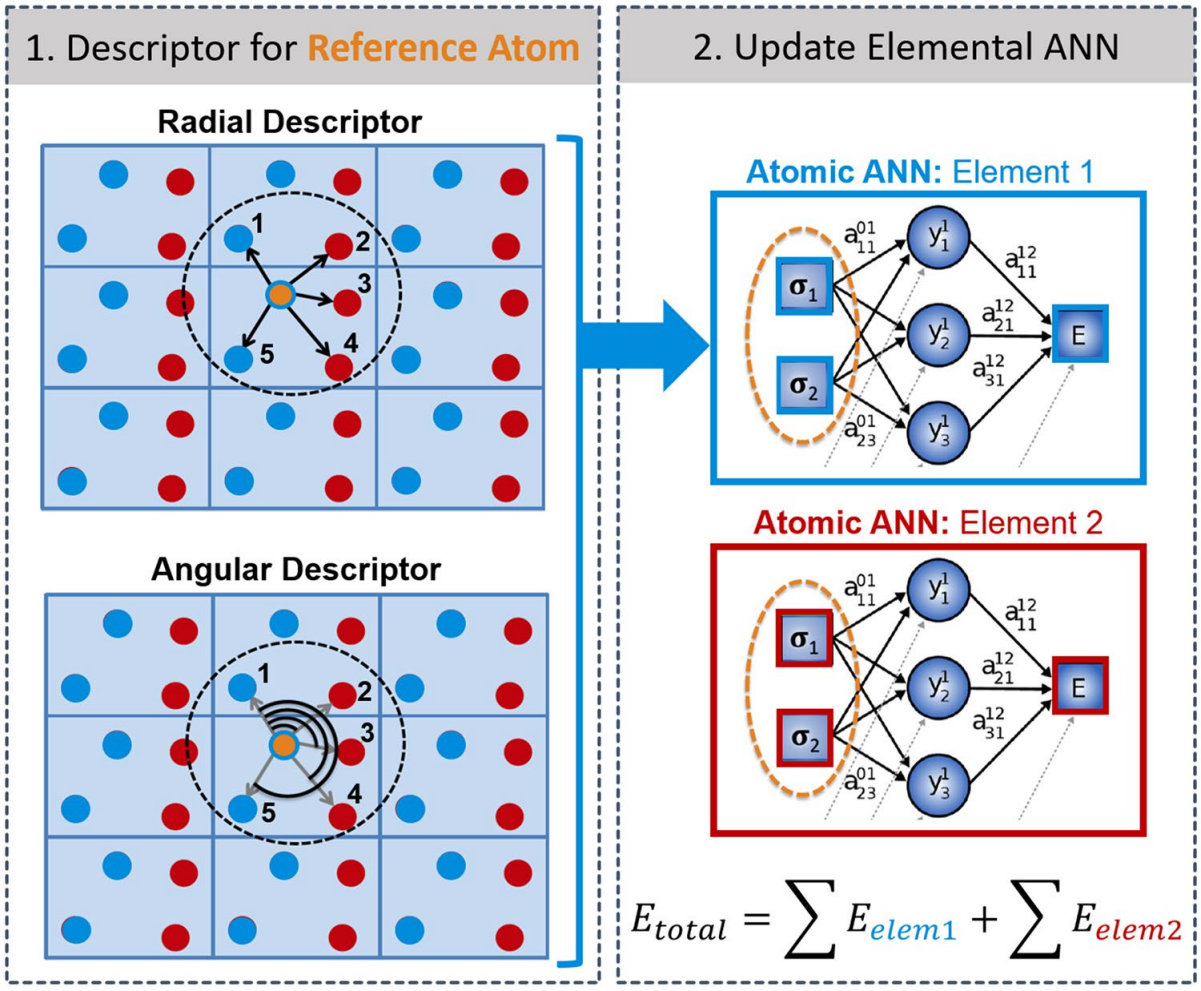
Schematic of radial and angular descriptors used for the representation of local atomic environments (left). The descriptor functions extract features that are used as input values for atomic energy ANNs. Separate ANNs for each atomic species (chemical element) are trained, so that the total energy of a binary material consist of two terms (right)

The descriptor introduced with the original method by Behler and Parrinello is based on an representation of the coordinates within the local atomic environments in *symmetry functions* [85, 95]. The symmetry functions and modified variants are commonly used as descriptors in public ANN potential implementations, such as ænet [127], AMP [128], ANI [129], TensorMol [108], and N2P2 [130].

**Fig. 6:**
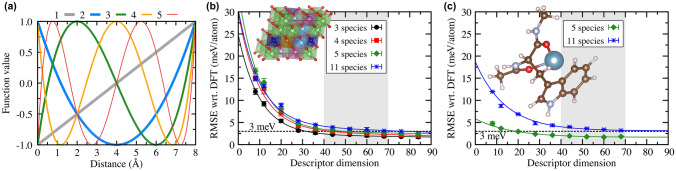
The Chebyshev descriptor (implemented in ænet [127]) enables the simulation of multicomponent compositions with many different chemical species. **(a)** Basis functions $\{\phi _{\alpha }\}$ of Eqs. (9) and (10) (Chebyshev polynomials) up to order 5 for a cutoff radius of 8.0 Å. The polynomial of order 0 is constant 1 and not shown. **(b)** and **(c)** show the accuracy of artificial neural network (ANN) potentials in terms of the root-mean-squared error (RMSE) compared to the QM reference method (DFT) as function of the size of the structural fingerprint (descriptor) for **(b)** an inorganic solid (${\hbox {Li}M\hbox {O}_{2}}$) with increasing number of chemical species (from the set Li, O, Ti, Ni, Mn, Sc, V, Cr, Fe, Co, and Cu) and **(c)** a data set with conformations of the 20 proteinogenic amino acids (5 chemical species: H, C, N, O, S; green diamonds) and their complexes with divalent cations (amino acid data taken from Ref. [131]). (Reproduced with permission from Ref. [132]. Copyright (2017), American Physical Society.)

Behler proposed two classes of symmetry functions [95], radial functions that capture the interatomic distances within the local atomic environment and angular functions that describe the bond-angle distribution. The symmetry functions have the general functional form

8 $$G_{\text{radial}}{ (\sigma_{i})}= \sum_{j\in \sigma_{i}} {g_{1}(R_{ij}) f_{\text{c}}(R_{ij}), G_{\text{angular}}(\sigma_{i})}={\sum_{j,k\in \sigma_{i}}} {g_{2}(R_{ij}, R_{ik}, R_{jk}) f_{\text{c}}(R_{ij}) f_{\text{c}}(R_{ik}) f_{\text{c}}(R_{jk})},$$

where $f_{\text {c}}$ is a cutoff function that smoothly goes to zero at the cutoff of the local atomic environment, and $g_{i}$ are parametrized functions designed to sample the distributions of bond lengths and angles, respectively. The dependence on the interatomic distance $R_{jk}$ may be omitted in $G_{\text {angular}}$. During the construction of an ANN potential, the number of symmetry functions and the parametrization of the functions $g_{1}$ and $g_{2}$ are meta parameters that have to be optimized. In addition to Behler’s original set of symmetry functions [85, 95], the ANN potential implementation ANI introduced a set of modified symmetry functions with slightly different definitions of the functions $g_{i}$ [129]. A schematic of the bond length and angle distribution within a local atomic environment is shown in Fig. 5.

The manual parametrization of $g_{1}$ and $g_{2}$ in the symmetry functions of Eq. (8) has advantages for ordered structures and molecular systems with well-known bonds and angles, but it complicates the construction of general ANN potentials when no such assumptions can be made. Recently, Li et al. proposed a formalism for the automatic optimization of symmetry function parameters based on pair-distribution functions [133].

As an alternative, Artrith, Urban, and Ceder developed a descriptor that is based on a formal expansions of the radial and angular distribution functions in an orthonormal basis set $\{\phi _\alpha \}$ [132]. For the radial distribution function (RDF) around atom *i*, this expansion can be written as

9 $$\text {RDF}_i(r) = \sum _\alpha c_\alpha ^{(2)} \phi _\alpha (r),$$

where the expansion coefficients $\{c^{(2)}_\alpha \}$ are invariant features of the local atomic environment and are given by

10 $$c^{(2)}_\alpha = \sum _{{{\mathbf {R}}}_j\in {}\sigma _i} \phi _\alpha (R_{ij})\, f_\text {c}(R_{ij})\, w_{t_j}.$$

The expansion of the angular distribution functions is completely analogous. Equations (9) and (10) introduce the basis functions $\phi _\alpha$ and an atom-type (chemical species) specific weight parameter $w_{t_j}$. Artrith et al. [132] chose Chebyshev polynomials as orthonormal basis set (see Fig. 6a), and the radial and angular distributions can be refined to arbitrary accuracy by including polynomials with increasing order without the need for manual parametrization. Faber et al. [121] previously employed a Fourier expansion.

The symmetry functions allow constructing a representation of the local structure but do not encode the chemical species, which is also needed for an accurate ANN potential. In the original multicomponent ANN potential method [96], the chemical species are distinguished with separate sets of symmetry functions for each combination of two (radial functions) and three (angular functions) chemical species. This approach results in an increase of the descriptor dimension and thus the computational effort with the number of atomic species, which makes it challenging to construct ANN potentials for more than a few chemical elements [86].

The Chebyshev descriptor [132] removes this scaling by introducing a weight parameter, $w_{t_j}$ in Eq. (10), that is different for each chemical species $t_j$. In fact, the descriptor contains two sets of expansion coefficients; the first set is evaluated without distinguishing between chemical species, i.e., for $w_{t_j}=1$, to represent *local structure* information. The second set of coefficients is evaluated with species-specific weights to capture differences in the *local chemistry*. By combining both local structure and chemistry, changes in the atomic positions and in the chemical species can be clearly distinguished. The dimension of the Chebyshev descriptor does not depend on the number of chemical species, and thus compositions with many chemical species do not result in any computational overhead [132]. Gastegger et al. later introduced species weights also to Behler-Parrinello symmetry functions in the *weighted atom-centered symmetry functions* method [134], though this approach does not include a separate structure descriptor. As seen in Fig. 6b–c, the accuracy that ANN potentials with the Chebyshev descriptor can achieve is not significantly affected by an increasing number of chemical species.

**Fig. 7:**
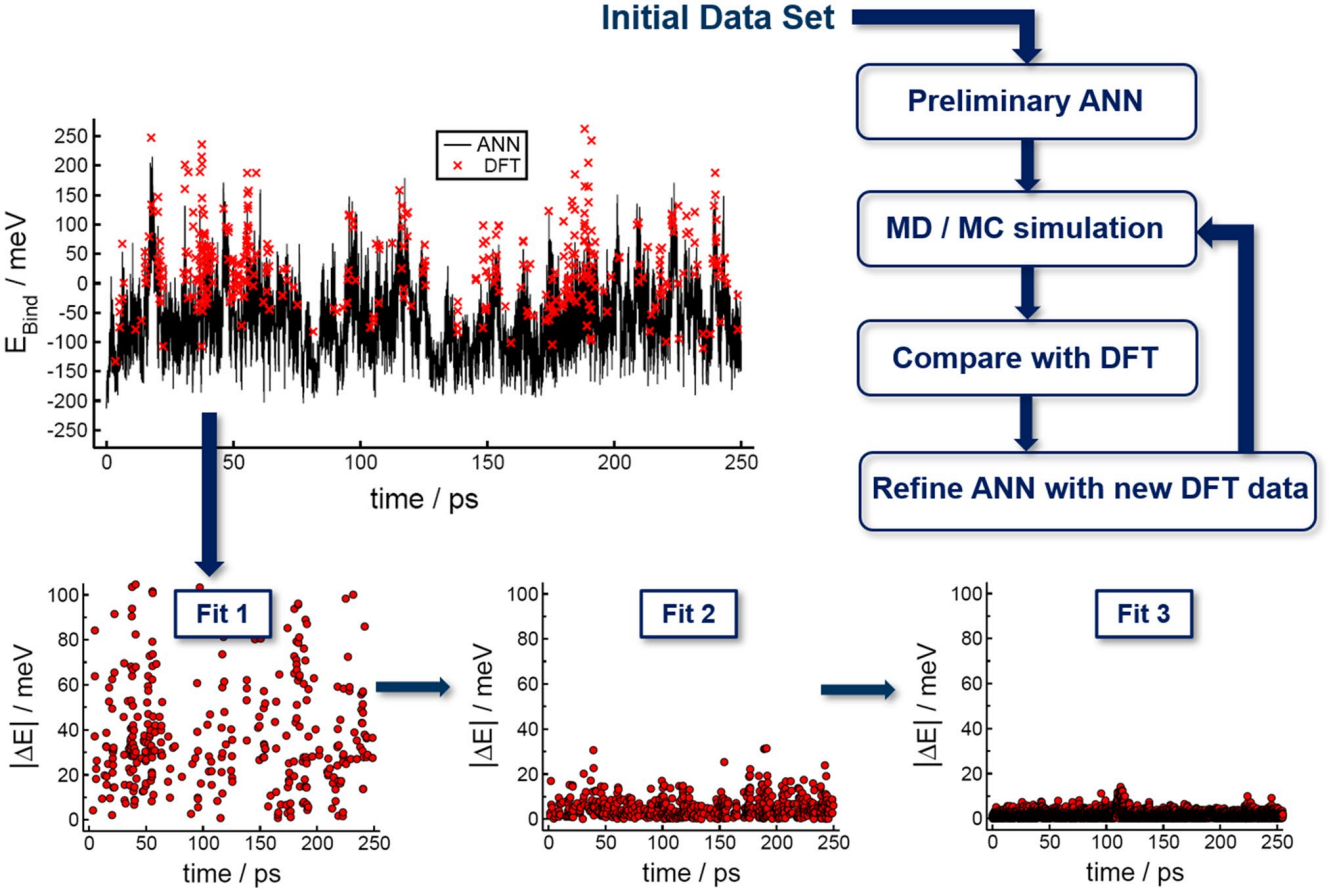
Illustration of the systematic construction of ML potentials through the refinement of the reference data set in an active learning setup. The error $|\Delta \text {E}|$, i.e., the difference between the reference DFT and the ANN energies, for structures obtained in MD simulations decreases upon each iteration, from Fit 1 to Fit 3, as the sampling of the configurational space improves. (Adapted with permission from Ref. [102])

### Training ANN potentials

The training of ANN potentials is the process of optimizing the weight parameters $\{a_i\}$ and $\{b\}$ in Eq. 1 for all artificial neurons. In the conventional ANN potential method, training on reference total energies from QM methods is most efficient, though approaches for the training of interatomic forces [130, 135] in addition to the energy have been developed. Chmiela et al. developed an alternative approach in which the forces are the only optimization target that uses an energy conservation criterion to avoid overfitting [136].

Irrespective of the training method, the reference data set is of critical importance for the transferability of ANN potentials. To guarantee complete reference data, active learning approaches are usually employed to systematically improve the data set [99]. A schematic of such an iterative refinement is shown in Fig. 7.

The principal idea behind active learning techniques is to make use of preliminary ANN potentials for the sampling of underrepresented structures. As such, oftentimes an initial data set is constructed based on chemical intuition, for example, by modification of ideal crystal structures or molecular geometries through scaling or deformation [127]. A preliminary ML potential is trained on this initial data and used in MC or MD simulations related to the eventual target application. A subset of the sampled structures is compared with the QM reference method, and if the discrepancy between the ANN prediction and the reference is too large, the structure is added to the reference data set. By repeating this procedure multiple times, the ANN potential becomes increasingly robust and transferable.

We outline here a basic active learning strategy but note that advanced techniques that improve the structure selection step are currently a very active field of research.

**Fig. 8:**
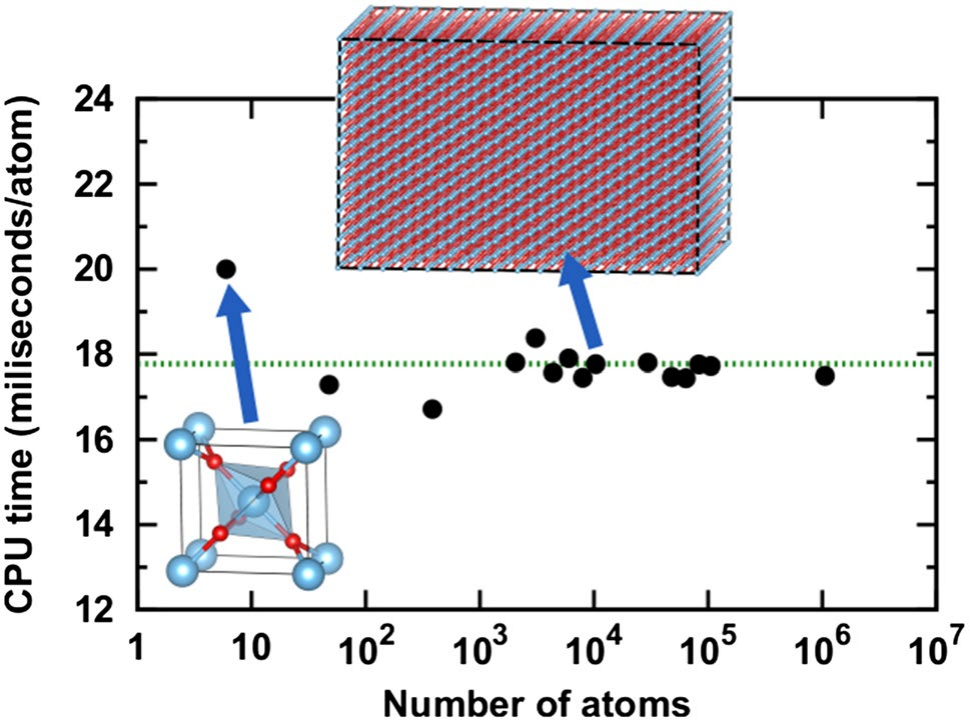
The computational complexity of ANN potentials scales linearly with the number of atoms. The plot shows the evaluation time per atom as function of the number of atoms for periodic ${\hbox {TiO}_{2}}$ structures with increasing size up to one million atoms. (Reproduced with permission from [127])

### Overview of MLP methods and implementations

Thanks to the decomposition of the total energy into atomic contributions, Eq. (2), the computational complexity of the ANN potential method scales linearly with the number of atoms and can be implemented in efficient computer code (Fig. 8). Robust and easy-to-use public implementations have been emerging over recent years. Since the field of ML methods for atomistic simulations is advancing rapidly and new software implementations, descriptors, ML approaches, and training frameworks are published at a high rate, we refer here to a collection of publicly available tools and databases that will be continuously updated to include the most recent developments in the field: https://github.com/atomisticnet/tools-and-data.

## Applications to industry

The previous chapter has summarized the methodological advances that now enable to apply ML approaches to complex molecular and materials systems under realistic conditions. In the remainder of this review we will discuss recent examples in which MLPs and ML models are used to accelerate the calculation of industrially relevant properties with focus on drug discovery and materials design. A selection of the obtained properties, compared to their corresponding reference values where available, is summarized in Table 1.

**Table 1:** Examples of properties calculated from machine learning (ML) potential simulations or using ML models based on quantum mechanical reference data compared to reference values, where available

Property	System	ML Prediction	Reference value	Year	Refs.
Drug discovery					
Reaction free energy	Glycine proton transfer	7.7 kcal/mol	DFT: 8.1 kcal/mol	2018	[137]
Reaction barrier	Glycine proton transfer	9.9 kcal/mol	DFT: 10.2 kcal/mol	2018	[137]
Solvation free energy	Acetic acid	$-\,$7.3 kcal/mol	DFT: $-\,$7.5 kcal/mol	2019	[138]
	Acetamide	$-\,$11.7 kcal/mol	DFT: $-\,$12.1 kcal/mol	2019	[138]
	Acetone	$-\,$3.9 kcal/mol	DFT: $-\,$4.3 kcal/mol	2019	[138]
	Benzene	$-\,$0.6 kcal/mol	DFT: $-\,$0.6 kcal/mol	2019	[138]
	Ethanol	$-\,$4.6 kcal/mol	DFT: $-\,$4.8 kcal/mol	2019	[138]
	Methylamine	$-\,$2.5 kcal/mol	DFT: $-\,$5.2 kcal/mol	2019	[138]
	Aqueous LiF pair	$-\,$231.5 kcal/mol	Exp.[139]: $-\,$232.9 kcal/mol	2020	[140]
Li-ion batteries					
	*Amorphous silicon anode*				
Li diffusivity	${\hbox {a}-\hbox {Li}_{x}\hbox {Si}}$ ($0.75<x<3.50$)	$10^{-14}$− $10^{-10}$ cm$^2$ s$^{-1}$	Exp.[141–144]: $10^{-14}$ − $10^{-10}$ cm$^2$ s$^{-1}$	2019	[145]
Activation energy	${\hbox {a}-\hbox {Li}_{x}\hbox {Si}}$ ($0.75<x<3.50$)	0.5 − 0.8 eV	N/A	2019	[145]
	${\hbox {a}-\hbox {Li}_{x}\hbox {Si}}$ ($0.02<x<0.06$)	1.21-1.46 eV	Exp.[146]: 1.38 − 1.46 eV	2020	[147]
	*Solid electrolytes*				
	Amorphous-${\hbox {Li}_{3}\hbox {PO}_{4}}$	0.55 eV	Exp.[148]: 0.58 eV	2017	[149]
	${\hbox {Li}_{10}\hbox {GeP}_{2}\hbox {S}_{12}}$	0.16 eV	Exp.[150]: 0.22 eV	2020	[151]
	${\hbox {Li}_{7}\hbox {La}_{3}\hbox {Zr}_{2}\hbox {O}_{12}}$	0.2 − 0.22 eV	Exp.[152]: 0.21 − 0.22 eV	2020	[151]
	*Cathode coating materials*				
	${\hbox {Li}_{2}\hbox {B}_{7}\hbox {O}_{12}}$	0.56 ± 0.05 eV	N/A	2020	[153]
	${\hbox {Li}_{3}\hbox {Sc}_{2}(\hbox {PO}_{4})_{3}}$	0.62 ± 0.04 eV	Exp.: [154] 0.65 eV	2020	[153]
	${\hbox {Li}_{2}\hbox {B}_{6}\hbox {O}_{9}\hbox {F}_{2}}$	0.79 ± 0.10 eV	Exp.: [155] 0.92 eV	2020	[153]
	LiCl	1.11 ± 0.13 eV	Exp.: [156] 0.83 eV	2020	[153]

*x* is the relative lithium content in the amorphous Li-Si alloys and varies during battery charge and discharge

### Drug discovery applications

In this section we discuss the use ML potentials and models for the prediction of properties that are relevant for drug discovery with the focus on two types of applications: the calculation of free energies and the prediction of spectroscopic properties.

A major challenge in calculating these properties lies in the complexity of the involved systems. The employed models have to be able to accurately describe small molecules, large molecular crystals and proteins interacting with small ligands in a solvent. These systems are governed by a diverse set of interactions between many different chemical elements involving diverse bonding types and (potentially) chemical reactivity. In contrast to simpler materials system, large units cells are required which makes it challenging to perform QM calculations. This is not only a problem for obtaining energies and forces required to perform MD or MC simulations but also for calculating other observables that are not (or only approximately) available from force fields. QM calculations in principle allow to calculate molecular dipoles, polarizabilities, and chemical shifts for simulating infrared (IR), Raman, and NMR spectra. Having access to these spectra allows to link experimentally observed spectral features to their corresponding molecular motion which in turn enables to identify molecular structures.

As discussed in the previous section, the application of MLPs to complex systems with diverse chemical environments has now become possible with new methodological improvements including more general descriptors for multi-component systems, automated training set generation, and force training. Even though these new approaches have just been developed (and continue to being extended) there are already several impactful applications of QM-based MLPs and ML models applied to bio-molecular systems relevant for the drug discovery process. Here we highlight some recent examples covering the investigation of chemical reactions in solution and solvation processes, the extension of force fields and semi-empirical QM/MM methods for simulations with improved accuracy, and the prediction of spectroscopic properties for the characterization of molecules and molecular crystals.

#### Reaction and solvation free energies

**Fig. 9:**
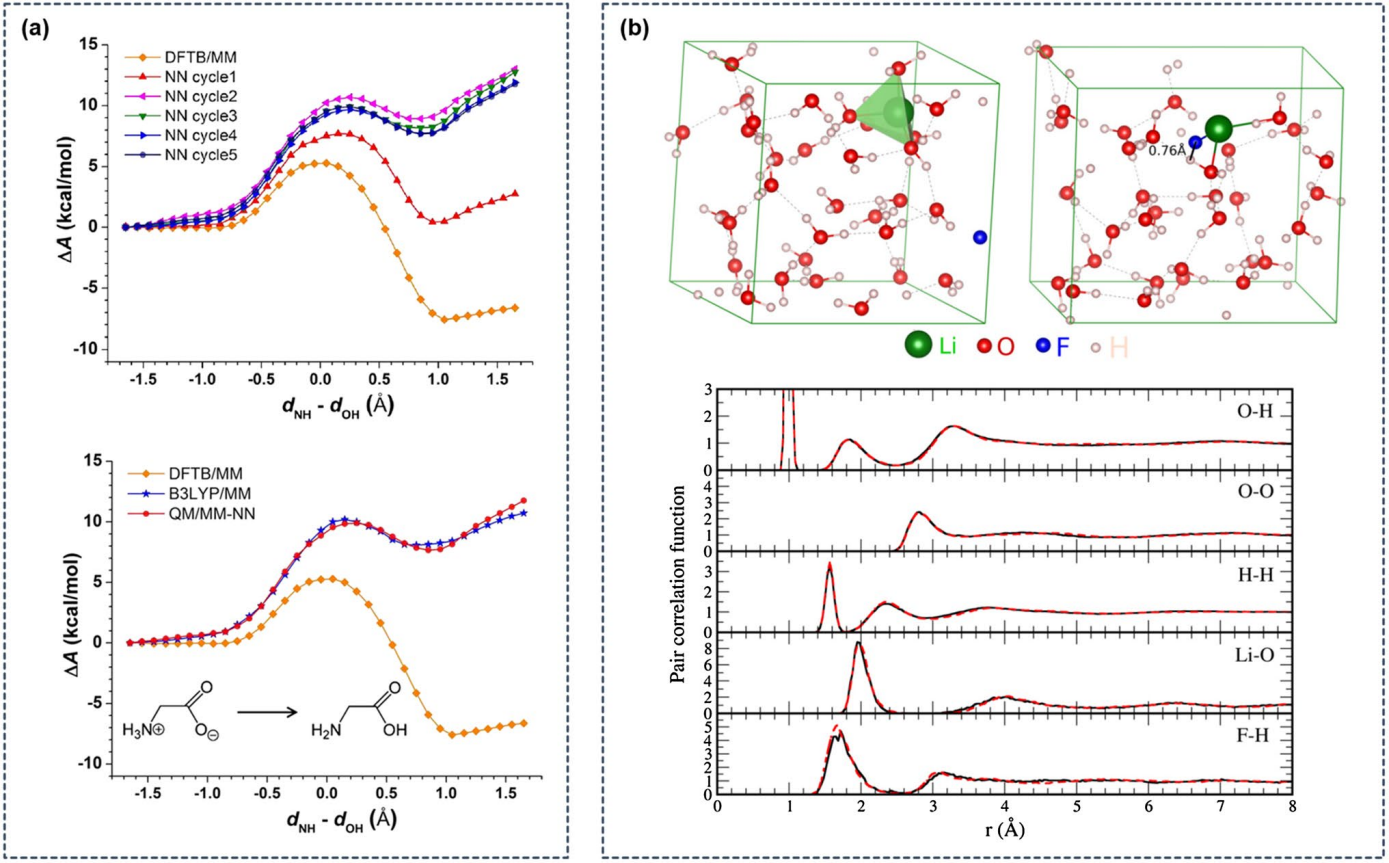
Machine learning simulations for free energy calculations: **a** Intramolecular proton transfer reaction of glycine in water by Shen and Yang [137] using a QM/MM-NN setup in which an MLP is iteratively trained (top) to represent the energy difference between a low-level (DFTB) and a high-level (B3LYP) QM method. In the final iteration (bottom) the MLP correctly predicts the zwitterionic glycine tautomer as the predominant form, improving on the inaccurate description with the low-level method. **b** Solvation free energy of LiF in water by Jinnouchi *et al.* [140] obtained from MLP-accelerated simulations trained on only the thermodynamic endpoints. The top panels show snapshots from thermodynamic integration simulations that correspond to the fully interacting system (left) and the system at small interactions (right), respectively. In the bottom panel pair-correlation functions of LiF in water obtained from the MLP (black line) are compared to results from QM simulations (red dashed line). A comparison of the ion solvation free energies is reported in Table 1

As discussed in the introduction, knowledge of binding free energies [22–24] allows to estimate relative binding affinities of a series of ligands and to rank them accordingly. Free energies are also central to understanding reaction mechanisms and transport processes. Solvation free energies determine the transport of a drug molecule to the target, which involves traveling through both aqueous media (blood) and lipophilic media (membranes) followed by desolvation before forming a ligand-protein complex [157]. The main challenges for obtaining reliable free energy values are insufficient sampling times and an inaccurate description of the PES.

Shen and Yang[137] employed ANNs to improve the accuracy of free energy calculations for two chemical reactions in solution, an S$_N$2 reaction and the intramolecular proton transfer reaction for glycine in water. Since chemical bonds are broken and formed during the process, a quantum mechanical description of the system for example within a QM/MM setup is required. To lower the computational effort, the QM part can be replaced by a semi-empirical (SQM) method such as scc-DFTB [158] which results in a more efficient but less accurate description of the system. As shown in Fig. 9a the neutral form of aqueous glycine is incorrectly predicted as the dominant one. To improve on that, the authors developed the QM/MM-NN approach in which the energy difference between the lower-level SQM method and the high-level QM method is predicted by an ANN potential. An earlier example of such a composite strategy in which an ML correction is added to a computationally efficient but less accurate QM method is the delta-machine learning approach by Ramakrishnan et al. [159]. An important ingredient of the approach by Shen and Yang is the use of an adaptive procedure in which an initial MLP is iteratively improved by new structures, selected when the ANN input variables are outside of their training set boundaries. Using the QM/MM-NN setup with the MLP correction term, in each iteration of the potential the description of the free energy along the reaction coordinate improves until with the 5th iteration it is closely aligned with the high-level QM result (see Fig. 9a and Table 1) and correctly predicts the zwitterionic form as having the lowest free energy. For the glycine system, the difference MLP leads to a total CPU time for the QM/MM-NN MD simulations that is only about 1–8% of the time required to perform simulations with the conventional QM/MM setup, yielding an increase in efficiency by a factor of 10–100. This comparison already includes the additional computational cost of running the QM reference calculations and training the MLP.

In a follow-up paper Zhang *et al.* [138] calculated solvation free energies for six small organic molecules (see Table 1) with an extended approach that addresses two challenges, (1) the identification of insufficiently sampled reference structures, and (2) the re-optimization process of the model after new structures were added to the training set. The selection criterion used in the previous work is based on the descriptor boundaries and by that has the potential to miss new data points that could lie inside the boundary region but be still very different from the current training structures. The authors therefore explored other approaches based on the energy range and different clustering algorithms. The training process was improved by using the component-wise gradient boosting algorithm [160] as a method to re-optimize the model with new data rather than each time training new models from scratch. These improvements enabled further time savings compared to the previous approach, requiring shorter simulations time and fewer additional structures for obtaining converged potentials. While in the extended approach a linear regression model was employed it will be interesting to see if an extension to more complex ML models such as ANNs can lead to further improvements.

Jinnouchi *et al.* [140] made use of a previously developed learn-on-the-fly ML approach [161, 162] to calculate solvation free energies of aqueous LiF ions at low computational cost. The solvation free energies of ions in water determine the properties of electrolyte solutions and greatly impact pK$_a$ values [163] and protein stability [164]. Ion solvation free energies can in principle be obtained from QM simulations [165] by methods like thermodynamic perturbation theory (TPT) [166] or thermodynamic integration (TI) [166, 167] but have large error bars. In an TI approach one can perform a coupling constant integration from a reference system comprising of the non-interacting ion pair in solvent to a system where all atoms fully interact (see Fig. 9b). The authors now employed a variant of the Gaussian Approximation Potential (GAP) approach [87] with the Smooth Overlap of Atomic Positions (SOAP) descriptor [88] for the on-the-fly generation of an MLP [161, 162] to speed up the TI simulations and obtain converged results. This approach employs self-learning to reduce the need for human intervention by using Bayesian inference to identify structures with high uncertainties which where then recalculated with the reference QM method and used to refine the MLP. A keys feature of the TI approach is that is requires only model training for the thermodynamic end points (the non-interacting and the fully interacting system). As shown in Table 1, the final free energy values obtained with the ML approach agree closely with the experimental values and also with results from QM simulations [163] while 10 times longer simulation times could be employed to reduce error bars and obtain converged values. Since the method is general and applicable to different systems it could be extended to obtain molecular solvation energies.

#### Spectroscopic techniques for structure characterization

**Fig. 10:**
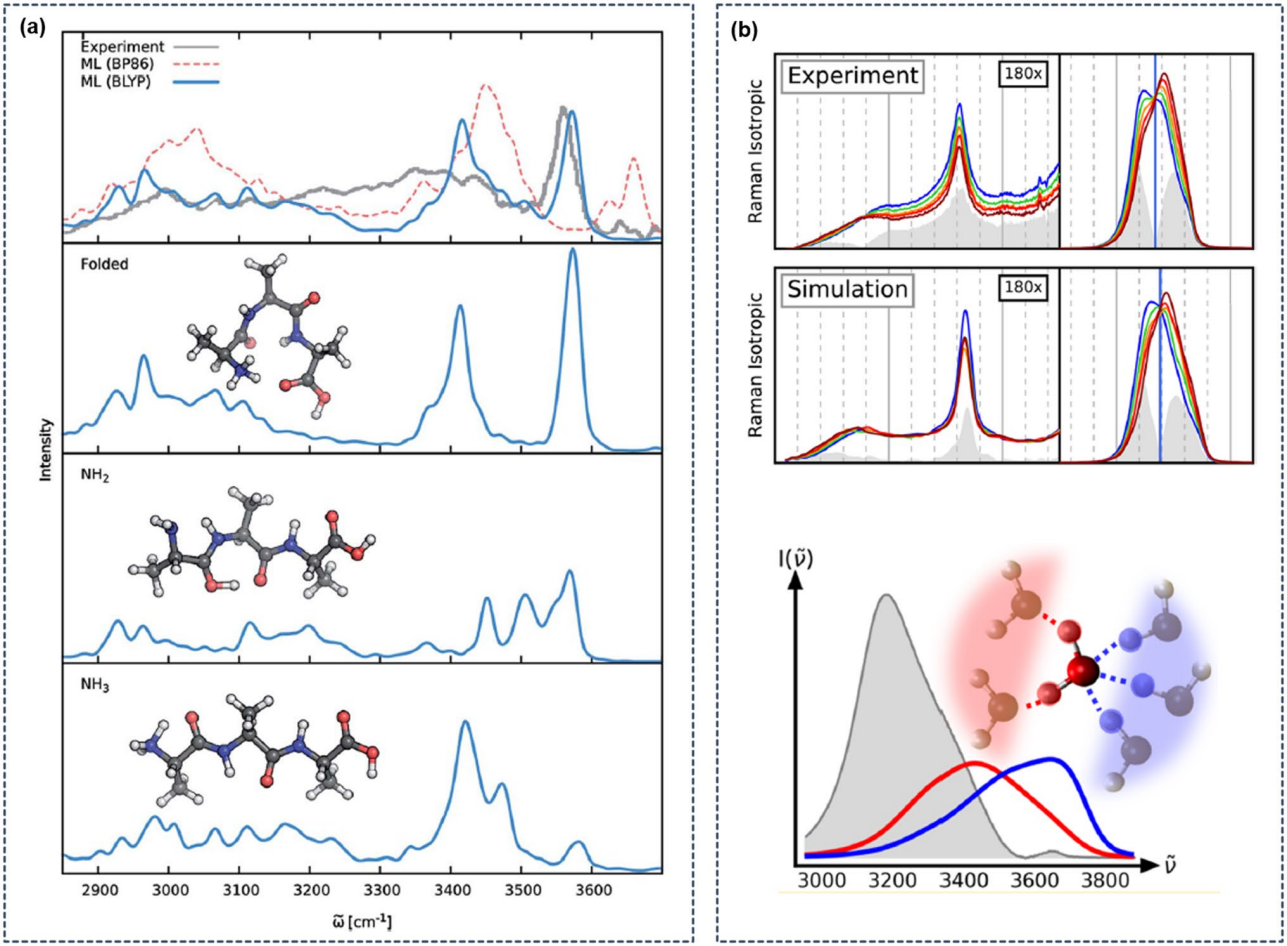
Machine learning prediction of spectroscopic properties: **a** IR spectrum of the protonated alanine tripeptide by Gastegger *et al.* [168] obtained from a composite ML approach in which the interatomic potential and the molecular dipoles are represented by individual ML models (Reproduced with permission from Ref. [168]—Published by The Royal Society of Chemistry). In the top panel, the calculated spectrum obtained from ML models representing two different QM methods (BP86 and BLYP) is compared to the experimental spectrum [169]. The bottom panels show spectral contributions from the three main conformers. **b** Temperature-dependent Raman spectra of liquid water by Morawietz *et al.* [170, 171] calculated from MLP simulations and compared to experimental measurements. As shown in the top panels (Reprinted with permission from Ref. [170]. Copyright 2018 American Chemical Society), MLP-based simulations are able to accurately capture subtle spectral features like the bimodal OH stretching region and allow to identify molecules in overcoordinated environments by linking vibrational motion to structural parameters (bottom panel, Reprinted with permission from Ref. [171]. Copyright 2019 American Chemical Society)

**Fig. 11:**
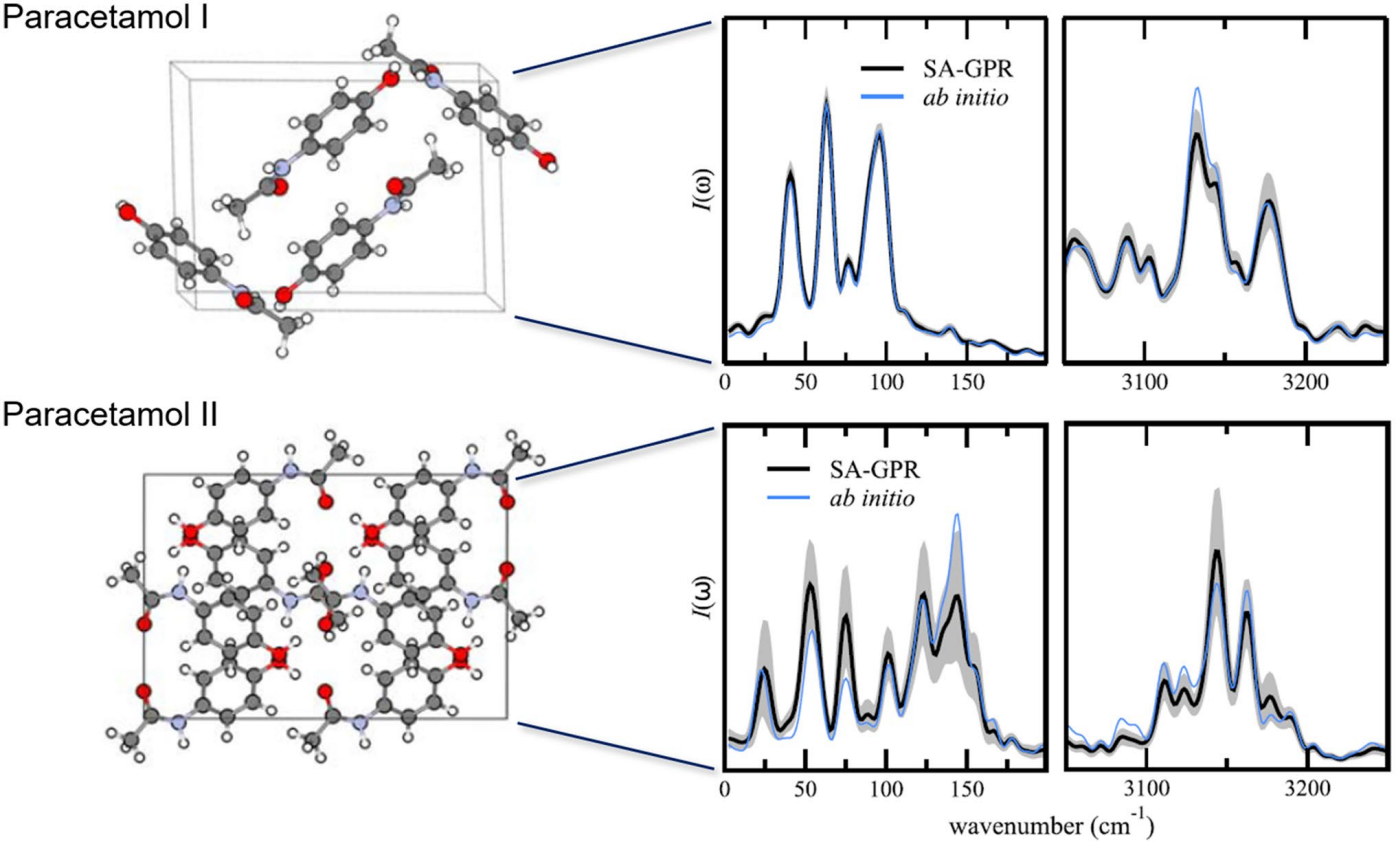
Machine learning prediction of spectroscopic properties: Anharmonic Raman spectra of the Paracetamol crystal in forms I and II by Raimbault *et al.* [172] calculated with an ML model (SA-GPR) of the polarizability tensor trained on form I only. The top panels show the low- and high-frequency parts of the Raman spectrum for form I compared to the reference QM results (ab initio). ML results were obtained from an ensemble of 16 models from which uncertainties have been estimated (shaded area). The results for form II in the bottom panels demonstrate the high transferability of the ML model which can accurately represent the overall lineshape of the unseen molecular crystal

Combining experimental measurements with QM calculations of infra red (IR), Raman, or NMR spectra is a powerful way to characterize the structure of molecular systems. Such combined approaches can for example help to identify the crystal structure of drugs that are available in form of molecular crystals which is the case for many marketed drugs. Understanding their crystalline form is crucial since it has an impact on several important properties such as stability, solubility, and bioavailability [173]. ML methods can help in two ways to improve the calculation of spectroscopic properties from atomistic simulations. They can (1) be used to represent the observables (dipoles, polarizabilities, NMR nuclear shifts) that are the ingredient for obtaining the spectrum and whose calculation by QM methods is often the computational bottleneck. Here, the structures for which the ML model predicts the spectrum are generated by performing a regular MM- or QM-based simulation. In approach (2), MLPs are trained to represent the PES of the system of interest and then efficiently generate the structures on which QM calculations are performed to calculate the spectroscopic property. Carrying out QM-calculations in such a post-processing step has the benefit that these calculations can be performed in parallel, as opposed to a purely QM-based approach in which a continuous trajectory has to be generated with a small time step and the observables are calculated on-the-fly. Approaches (1) and (2) can also be combined in a setup where the simulation is performed by an MLP and an additional ML model is used to represent the QM-trained spectroscopic observable. Here we discuss a number of recent examples in which ML approaches are applied to obtain spectroscopic properties for organic molecules in the gas phase, hydrogen-bonded liquids, and molecular solids.

IR spectra for bio-molecular systems are often obtained from static calculations with a normal mode analysis based on the harmonic approximation, thus neglecting important anharmonic and temperature effects. QM-based simulations allow to include these dynamic effects (and also reactive proton transfer events) but at high computational costs. A composite ML approach for the calculation of anharmonic IR spectra was developed by Gastegger et al. [168] in which the need for explicit QM calculations is fully circumvented by combining MLPs with an ML model to represent molecular dipoles. They employed ANNs to represent the PES, making use of an adaptive scheme for selecting new structures and training on atomic forces [174] which allowed the use of a small number of QM training points ($\sim$700 for the alanine tripeptide). To be able to train the MLP on large systems, a fragmentation approach was used in which large molecules are divided into smaller fragments for which reference QM calculations are more feasible. The molecular dipole moments were modelled by another ANN representing environment-dependent atomic charges. Since atomic charges are no observables, there is no unique way to calculate them. The solution used by the authors was to use the *total* dipole moment and molecular charge of the entire system as the target property. Equivalent to the atomic energies which are the output of atomic ANNs and in sum give the total energy of the system, the atomic charges can be seen as latent variables. The ML model that represents the environment-dependent charges therefore acts as a data-driven partitioning scheme without any constraints other then to match the target. Among other organic molecules, the protonated alanine tripeptide was used as test system to evaluate the ability of the combined ML approach to describe anharmonic, conformational, and dynamic effects, including proton transfer events, that all contribute to the IR spectrum (see Fig. 10a). While it was found that the resulting IR spectra show a strong dependence on the QM reference method, the efficiency of the ML approach (with timings of 1 hour instead of > 100 days on a single CPU for obtaining the full spectrum) allows to quickly benchmark different QM methods to find the most suitable one for the system at hand. The authors also suggested that ANN-learned atomic charges could not only be used to obtain vibrational spectra but also for the augmentation of classical force fields, a route that was taken in the following application.

Kato *et al.* [175] constructed ML models to predict accurate charges for three proteins based on fragment molecular orbital (FMO) calculations. Commonly used force fields use fixed atomic charges and therefore neglect electronic polarization. Since the force field charges cannot adjust to a changing environment the description of the molecular recognition process between a protein and a ligand molecule might suffer. To address this issue, the authors trained an ANN model to learn atomic partial charges from QM calculations that take into account electronic polarization. They faced the challenging task to perform QM reference calculations for three complete proteins (polyQ10, Trip-Cage, and BRD2) containing up to 111 amino acids. Similar to the work by Gastegger et al. this was addressed by employing a fragmentation approach with the FMO method. Element-specific ANNs using atom-centered symmetry functions where then trained to learn atomic charges from restrained electrostatic potential (RESP) calculations. In future applications, ML charge models could be combined with force fields to develop simulation methods that take into account polarization effects for the improved description of protein-ligand interactions. Energies and atomic charges from FMO calculations of 1074 proteins were made freely available by the authors.

Returning to ML applications for the simulation of vibrational spectra, Morawietz et al. [170, 171] simulated temperature-dependent Raman spectra for liquid water using MLPs. They initially employed a variant of approach (2) in which trajectories at different temperatures where generated from ML-based MD simulations. QM calculations were then performed on these structures to obtain the polarizabilities required to calculate Raman spectra. As shown in Fig. 10b (top), these spectra accurately reproduce experimental measurements across the full liquid temperature range. In a second step, the authors bypassed the use of QM calculations and used the vibrational density of states (VDOS), obtained from the atomic velocities, as a proxy for the vibrational Raman features. In an combined effort with experimental decomposition techniques they made use of the VDOS to identify the structural origin of subtle vibrational features in the Raman spectrum. This analysis could for example identify the vibrational fingerprints of molecules residing in over-coordinated hydrogen-bond environments, species that play an important role in the transport of protons through membranes and the coordination of hydrophobic groups (see Fig. 10b, bottom).

In a complementary approach, Raimbault et al. [172] predicted anharmonic Raman spectra of paracetamol using QM simulations to generate the trajectories and an ML model to predict polarizabilities. They compared different GPR methods to learn polarizability and susceptibility of molecules and molecular crystals for reference data from QM calculations using density-functional perturbation theory (DFPT) calculations. DFPT results for anharmonic vibrational Raman spectra of molecular crystals were taken from prior work and made available in the NOMAD database [176, 177]. DFPT calculations are typically four times more expensive then evaluating the forces during an MD simulation [177]. A symmetry-adapted GPR version (SA-GPR) [178] was found to be most suitable for describing tensorial properties such as polarizabilities. The SA-GPR approach has been also successfully applied to the prediction of Raman and IR spectra for liquid water and ice based on path integral MD simulations that include nuclear quantum effects [179]. Using an ensemble of 16 ML models to estimate uncertainties, Raimbault et al. applied their approach to calculate the Raman spectrum of two crystal forms of paracetamol (see Fig. 11). Impressively, the ML model trained only on crystal form I is able to accurately predict the spectral lineshape for form II, even though the low-frequency modes that correspond to the intermolecular interactions vary considerably between the two forms. The high degree of transferability demonstrates the benefit of using a local approach in which total polarizabilities are decomposed into atom-centered contributions based on local environments. While this approach still relies on QM simulations, we expect to see an increase in the number of “ML-only” approaches (as in Ref. [168]]) where MLPs are used to perform MD simulations and ML models (for example based on GPR) represent observables like dipole moments and polarizabilities to obtain accurate spectra with greatly reduced computational costs.

In the final example by Paruzzo et al. [180], NMR chemical shifts for molecular crystals were predicted by ML models based on the GPR approach. Chemical shifts are key data for determining structure and dynamics of bio-molecular systems and can for example help to identify the protonation state of enzyme active sites [181]. While many empirical tools have been developed to aid in the the assignment of experimental NMR spectra they are often optimized for a small subset of systems and neglect dynamical effects. Calculating chemical shift with QM methods [182, 183] has a more general validity for different chemical environments [184]. Combining QM calculations with NMR measurements enable chemical shift-based crystallography for validating the structure of molecular solids [185, 186]. Paruzzo et al. employed a GPR framework with the SOAP kernel [88] to learn DFT chemical shifts for structures from the CSD database [187] with estimated uncertainties based on a previously introduced resampling scheme [188]. The ML model was trained on 500 structures randomly sampled from a CSD subset containing 61,000 structures that are small enough (<200 atoms) to perform QM calculations and then applied to calculate chemical shifts of six molecular crystals comprising of up to 1500 atoms. The authors then demonstrated that, without making use of experimental chemical shifts, their method is accurate enough to correctly determine the structure of two molecular solids: cocaine and the drug AZD8329. This application again exemplifies the benefit of employing local ML models: they can be transferable to larger systems without loss of accuracy and in addition scale linear with system size. Calculating the full set of chemical shifts for six molecular crystals took a few minutes with the ML model which, for the largest model, is a speed-up of a factor of 10$^6$ compared to a direct QM approach. The ML tool to predict NMR chemical shifts for the elements $^1$H, $^{13}$C, $^{15}$N, $^{17}$O and $^{33}$S is publicly available [189].

### Materials discovery applications

In correspondence to drug discovery applications, the application of ML models to materials discovery has also seen a steep rise of research activity during the last decade, owing to the availability of methods, public implementations, and increased computer power [190]. In this section, we review some of the recent successful applications in the area with a focus on inorganic solid materials.

#### Phase diagram predictions

Before considering any other properties of a potential functional material, the first requirement for computational materials design is the ability to predict whether a hypothetical compound is stable. Predicting the products of organic synthesis (such as drugs) requires knowledge of reaction kinetics. In contrast, the stability of inorganic solids is mostly governed by thermodynamics [191]. As such, in good approximation, predicting the likely stability of a novel material is equivalent with predicting the thermodynamic phase diagram. This approximation can be further improved by considering also the kinetics of nucleation, e.g., by modeling the nucleation and growth of inorganic phases.

**Fig. 12:**
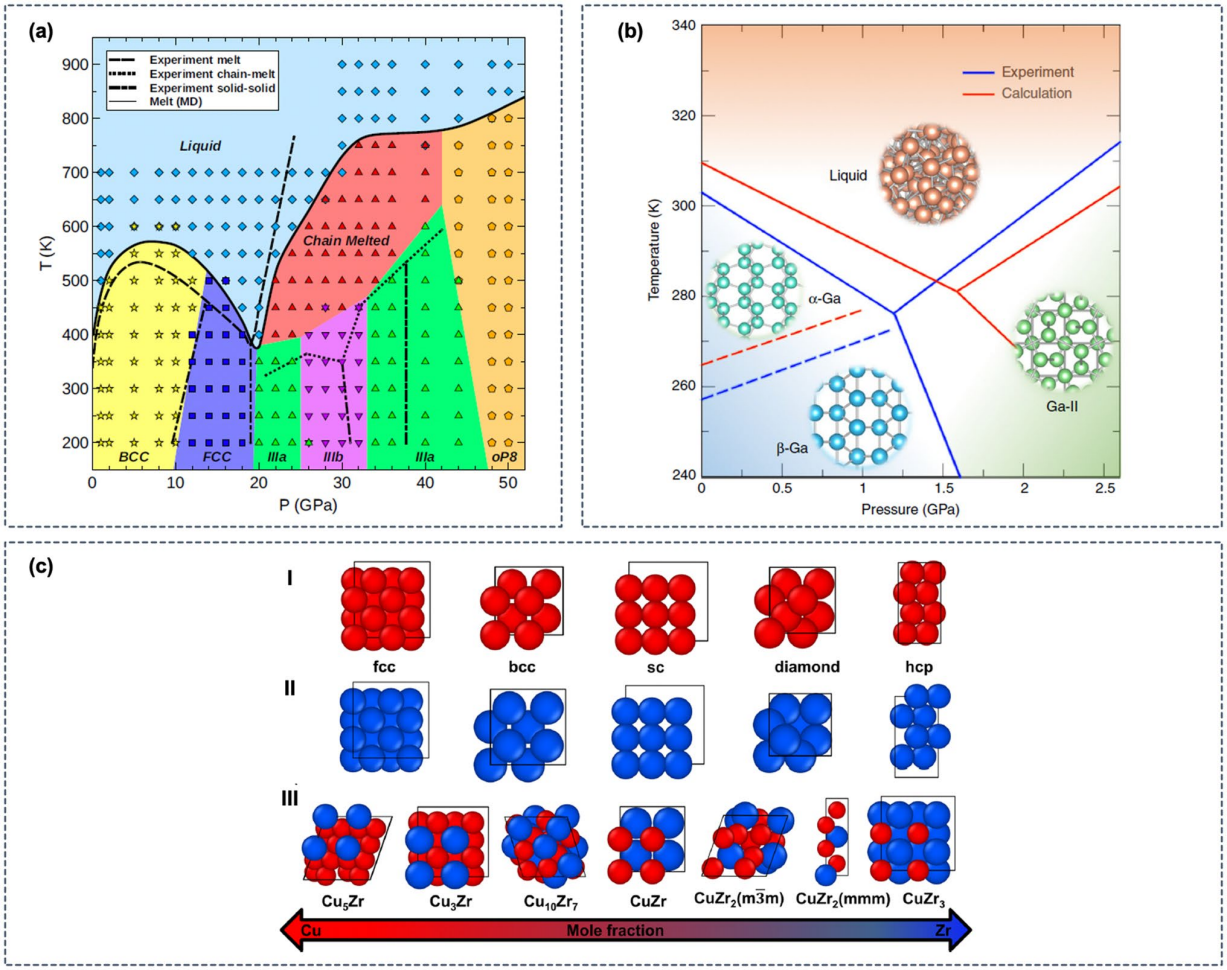
ML-based simulations for the exploration of phase diagrams of inorganic materials. **a** Temperature and pressure dependent phase diagram of potassium obtained from MD simulations using an ML potential [192]. Each point in the figure represents the result from an individual ML-based MD simulation in the *NVT* statistical ensemble. Symbols distinguish between different equilibrium phases. (Reproduced with permission from [192]). **b** Phase diagram of gallium nucleation from the melt using metadynamics MD simulations with an ML potential [193]. The predicted phase diagram (red lines) is compared to the experimentally measured phase diagram (blue lines). **c** Crystal structures of the CuZr alloys and of the Cu and Zr constituents used for training of an ANN potential by Andolina et al. [194]. The ANN potential trained on the crystalline phases was shown to predict the properties of the amorphous CuZr alloy with remarkable accuracy

Brute-force atomistic calculations of phase diagrams are challenging because the time scales on which phase transitions occur are often not achievable with QM simulation methods. Here, ML potentials can be used as a drop-in replacement in some cases. For example, Morawietz et al. simulated the melting of ice with MD simulations using ANN potentials [114], revealing the importance of vdW interactions for a correct description of the mechanism of the phase transition. Robinson et al. also used ML potential based MD simulations to determine the phase diagram of potassium as a function of the pressure and temperature [192], which exhibits a complex chain-melted phase that had previously not been characterized in detail. The MD simulations by Robinson et al. were initialized in the expected ground-state phase for a given pressure at 200 K, and MD simulations in the *NVT* statistical ensemble were used to simulate phase transitions with temperature. The resulting phase diagram is shown in Fig. 12a.

While the process of melting is challenging to model, the reverse, i.e., crystallization from the melt, typically occurs at even longer timescales owing to nucleation barriers. In few cases, rapid crystallization can be modeled with direct MD simulations. Sosso et al. investigated the fast crystallization of GeTe, a phase-change compound, from the supercooled liquid using ML potential MD simulations and identified the atomic-scale mechanism responsible for the rapid nucleation rate [195]. The same system was also investigated by Gabardi et al.  who observed the nucleation of crystalline GeTe in 3 ns long melt-quench MD simulations with an ANN potential [196], finding that a crystallization mechanism similar to that in supercooled liquids can be achieved.

For many materials, phase transitions between solid phases or crystallization cannot be modeled with direct MD simulations as the time scale remains inaccessible despite the speed-up from ML potentials. In such cases, ML potentials in combination with accelerated MD techniques, such as the metadynamics approach by Parrinello and coworkers [54, 197], have been successfully employed. Behler and Parrinello modeled the polymorphic phase transitions in elemental Si using metadynamics [198], and a similar approach was used by Eshet et al. for the construction of the $P-T$ phase diagram of elemental sodium [199]. Bonati and Parrinello investigated crystallization of silicon from the melt with well-tempered metadynamics [197] using an ANN potential [200], identifying a single collective variable derived from the Debye structure factor to steer the crystallization. A related approach was employed by Niu et al. for the calculation of the phase diagram for gallium nucleation from the melt [193]. Gallium exhibits a complex phase behavior owing to the mixed covalent and metallic bonding, making the element a challenging benchmark case for phase diagram calculations. As seen in Fig. 12b, the phase diagram predicted by accelerated ML potential MD simulations is in excellent agreement with the experimental reference, demonstrating that ML potentials are sufficiently flexible to capture the complex atomic interactions of elemental gallium.

ML potentials have also been used for the modeling of polymorphism and phases with variable compositions in compounds that consist of multiple chemical species. In two separate studies, Artrith and coworkers showed that an ANN potential can accurately reproduce the stability of different ZnO [96] and ${\hbox {TiO}_{2}}$ [127] polymorphs. Kong et al. used ANN potentials and an ML-augmented sampling technique to determine the phase diagram of CoO phases with varying Co:O ratio [201]. ANN potentials have been also used to model multicomponent alloys with varying composition, such as the AuCu alloys, both in the bulk and in nanoparticles [202, 203].

Apart from crystalline phases, the increased efficiency of ANN potentials compared to DFT calculations makes modeling disordered or amorphous phases accessible, which generally require larger structure models than crystal structures. Artrith et al. employed a combination of an evolutionary algorithm and an ANN potential to determine the phase diagram of the amorphous LiSi alloys [204]. In this study, the amorphous phase was explicitly sampled and characteristic structural motifs were included in the reference data set for the ANN potential training. Recently, Andolina et al. [194] trained an ANN potential on the crystalline CuZr alloy phases (Fig. 12c) and demonstrated that the resulting potential can accurately predict the properties of amorphous CuZr phases as well, which is a remarkable display of the transferability that ML potentials can achieve.

**Fig. 13:**
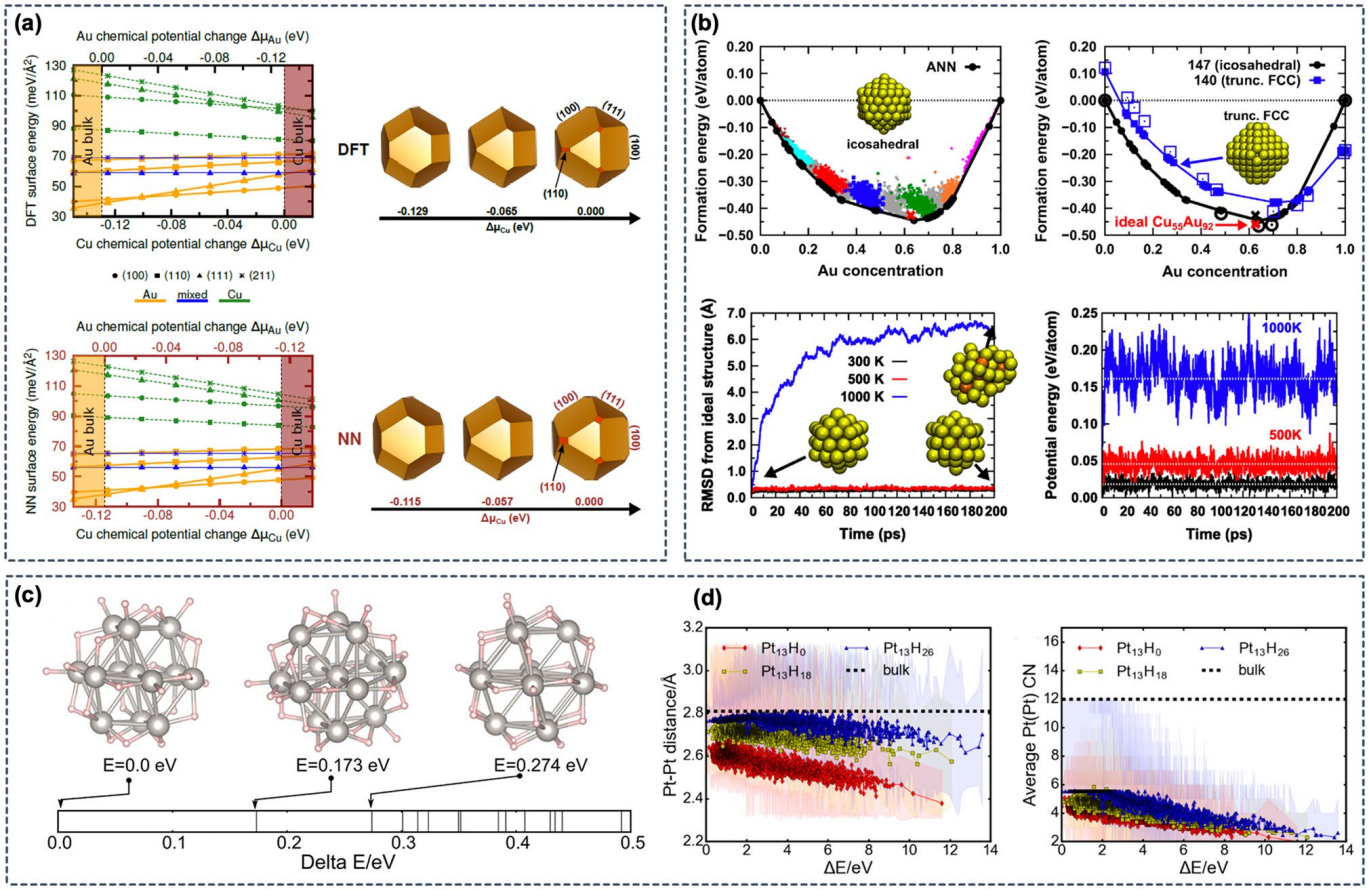
ML potential simulation of catalyst materials: **a** surface phase diagrams of the of low-index surfaces of the ${\hbox {Cu}_{2}\hbox {Au}_{2}}$ alloy with different terminations (Au, Cu, and mixed) as function of the Au/Cu chemical potentials, as predicted by DFT (top) and by an ANN potential (bottom). Symbols denote different facets, and surface terminations are indicated by line types and colors (yellow = Au terminated, blue = mixed, green = Cu). Exemplary Wulff constructions corresponding to three different chemical potentials are also shown. (Reproduced with permission from Ref. [202]) **b** Formation energies and convex hull construction for CuAu nanoparticles with 55 atoms. Different colors and point sizes indicate different chemical potentials used in grand canonical ($\mu {}VT$) MC simulations. (Reproduced with permission from Ref. [203].). **c** Low-energy structures of Pt nanoparticles in hydrogen atmosphere. The energies of the particle structures are shown relative to the most stable configuration. Statistics of the Pt-Pt nearest neighbor distances and the average Pt coordination number as function of the relative energy are shown in panel **(d)**. (Reproduced with permission from Ref. [205])

#### Properties of catalyst materials

The design and discovery of novel materials for heterogeneous catalysis is an area of great relevance for the chemical industry [206], and QM calculations for the computational prediction of the properties of catalyst materials are well established [207]. However, most computational studies make use of simplified catalyst models, such as single-crystal surfaces in vacuum, whereas catalytically active sites may in reality depend on the environment and on defects in the catalyst material. The greater computational efficiency of ML potentials has enabled the modeling of more realistic catalytic conditions and materials in recent years. Here, we focus again on applications of ANN potentials. Other ML applications for catalysis have recently been reviewed by Goldsmith et al. [208] and by Kitchin [209].

ML potential simulations have been of particular use for the modeling of non-idealized catalyst structures. For example, Artrith et al. constructed an ANN potential for the simulation of ZnO-supported Cu nanoparticles [100], the catalyst for methanol synthesis [210, 211], and investigated the dynamic structure changes of the catalyst at 1,000 K using MD simulations. Such large-scale MD simulations would not have been possible with first principles QM methods, and conventional interatomic potentials would not have been able to capture the mixed metallic and ionic bonding in the Cu/ZnO interface region.

Even unsupported catalyst nanoparticles are often beyond the length-scale limit of QM methods, especially when extensive sampling of atomic configurations is needed. Artrith and Kolpak showed that ANN potentials trained on surface structures and cluster configurations of CuAu alloys can reproduce the surface phase diagrams and Wulff shapes of the different alloys as a function of the chemical potentials (Fig. 13a) [202]. These ANN potentials were then used in large-scale MC simulations to determine low-energy atomic orderings in nanoparticles with up to 6500 atoms and in AuCu clusters and surfaces in contact with water, which showed the strong impact of water on the alloy surface termination and could explain the catalytic activity of CuAu nanoalloys for ${\hbox {CO}_{2}}$ reduction [202]. The same authors also employed ANN potentials to investigate the temperature-dependent dynamics of CuAu nanoparticles in grand-canonical ($\mu {}VT$) MD simulations [203]. The phase diagrams and the stability region of icosahedral CuAu nanoparticles compared to nanoparticles with truncated face-centered cubic shape are shown in Fig. 13b. Kolsbjerg et al. demonstrated for small ${\hbox {Pt}_{13}}$ clusters how a combination of an ANN potential and an evolutionary algorithm can be used for the search for low-energy cluster structures [212], finding that a thermal ensemble of low-energy structures provides a better description for the catalyst than the zero temperature ground state structure alone. Sun and Sautet also used an evolutionary optimization strategy coupled with an ANN potential to determine the structures of Pt nanoparticles in hydrogen-rich atmosphere [205], which revealed a complex interplay of the Pt particles with the hydrogen gas resulting in a rich distribution of thermally accessible metastable Pt nanoparticles with very different properties. Example particle configurations and statistics of the Pt-Pt nearest-neighbor distribution and Pt coordination numbers (CN) are shown in Fig. 13c–d.

#### Properties of battery materials

**Fig. 14:**
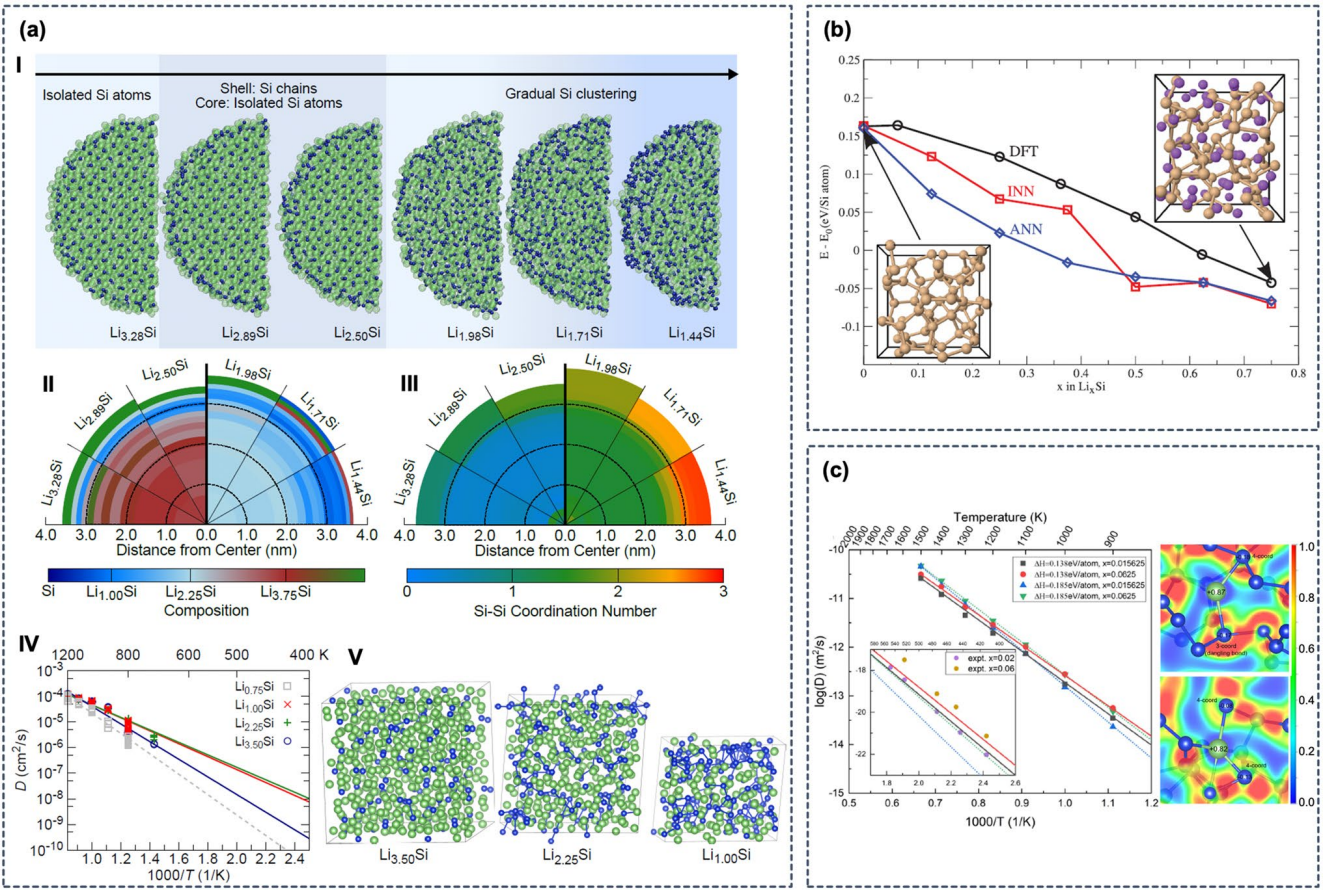
Lithium transport in amorphous silicon anodes for lithium-ion batteries. **a** Atomic structures of ${\hbox {Li}_{x}\hbox {Si}}$ alloy nanoparticle during delithiation (battery discharge) [145]. The change of the composition in the core of the ${\hbox {Li}_{x}\hbox {Si}}$ nanoparticles is shown in subfigure (a.I), and the change in the Si coordination numbers are shown in (a.II and a.III). Panels (a.IV) and (a.V) show an Arrhenius plot with the temperature-dependent lithium diffusivity in bulk amorphous LiSi alloys and representative bulk structures for different Li:Si ratios, respectively. **b** Formation energies of amorphous LiSi structures as predicted by two different ANN potential approaches (ANN and INN, implanted neural networks) compared to the DFT reference energies [213]. **(c)** Arrhenius plot for Li diffusion in different amorphous silicon structures (left) and visualization of the electron localization function (ELF) for different structural motifs in the amorphous LiSi, Li bonding to an undercoordinated Si atom (top) and Li bonding to a fully coordinated Si atom (bottom). The numbers indicate the Bader charges of the Li and Si atoms. (Reproduced with permission from reference [147])

The second major class of functional materials that has been investigated using ML potentials are materials for lithium ion batteries. Lithium ion batteries (LIB) consist essentially of two electrodes, the cathode and anode, that are submerged in or separated by electrolyte and are in contact with an external circuit. When an LIB is discharged (i.e., the battery is used), ${\hbox {Li}^{+}}$ cations are shuttled from the anode through the electrolyte to the cathode, and simultaneously electrons are released from the anode, perform work while they travel along the external circuit, and eventually arrive at the cathode as well. This process is reversed when the LIB is charged.

First principles QM calculations are widely used for the calculation of many properties of LIBs, such as the voltage and the electrochemical or thermal stability of the components [8]. However, QM based modeling is most practical for crystalline materials, although both non-crystalline electrode and electrolyte materials are of great relevance for LIBs. This limitation is especially significant for the investigation of Li transport in electrodes, the electrolyte, and interface regions, which can become rate limiting in LIBs. Recently, ANN potentials have enabled the simulation of Li diffusion in non-crystalline phases that had previously not been accessible.

**Fig. 15:**
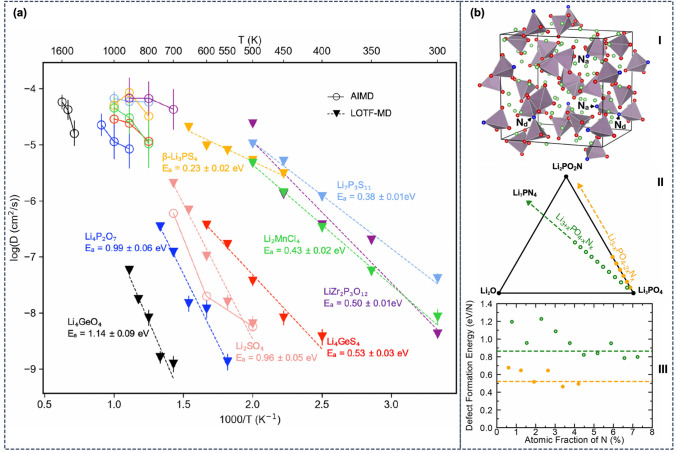
Machine learning simulations for solid-state batteries: **a** Arrhenius plot with Li diffusivities obtained from ab initio MD (AIMD) simulations using a learning-on-the-fly (LOTF) ML potential based on the MTP method [153]. The ML potential simulations make low temperatures accessible that are closer to room temperature, whereas conventional AIMD simulations are limited to very high temperatures that are not relevant for battery operation. (Reproduced with permission from Ref. [153]) **b** Representative structure (I) and DFT phase diagram (II) of LiPON near-ground-state crystal structures [214]. Two different composition lines for nitrogen doping are indicated in yellow (Li replacement) and green (Li addition), respectively. Panel (III) shows the corresponding defect formation energies for nitrogen doping, as calculated with ANN-potential augmented sampling and DFT calculations. All defect structures are predicted to be unstable with respect to decomposition into ${\hbox {Li}_{2}\hbox {O}}$, ${\hbox {Li}_{3}\hbox {PO}_{4}}$, and ${\hbox {Li}_{2}\hbox {PO}_{2}\hbox {N}}$, showing that amorphous LiPON is metastable. Nitrogen doping via Li replacement is thermodynamically favored over doping with Li addition. (Reproduced with permission from Ref. [214])

For example, nanostructured amorphous silicon (a-Si) is a promising anode material for the next-generation of LIBs but its reaction with lithium has not been well understood on the atomic scale [215]. Artrith et al. employed a combination of ANN potentials and an evolutionary algorithm to model electrochemical amorphization and to sample the phase diagram of the amorphous LiSi alloys [204]. The resulting ANN potential (based on a QM database reported in Ref. [216]) was subsequently used by the same authors to investigate Li transport in realistic LiSi nanoparticles containing up to 12,000 atoms including the amorphization and the change of the Li diffusivity upon delithiation (Fig. 14aI–III) [145]. One conclusion from these simulations is that the distribution of Si atoms within the structure strongly affects the Li diffusion, and Li rich regions are beneficial for Li diffusion (Fig. 14aIV–V). Onat et al. also trained ANN potentials for the modeling of amorphous LiSi structures [213]. The authors proposed an *implanted neural network* (INN) approach, in which the ANN potential is first pre-trained on the individual components (Li, Si) before it is used for the amorphous LiSi alloys (Fig. 14b). Li transport in the amorphous LiSi alloys was also investigated by Li et al. using ANN potential based MD simulations [147], also finding a strong dependence of the Li diffusivity on the local Si environment. Using the structure models from ANN potential simulations, the authors performed an electronic-structure analysis of the bonding in the atomic structures with DFT, finding that undercoordinated Si atoms interact more strongly with Li atoms and can impede Li diffusion (Fig. 14c), which is in agreement with the observations by Artrith et al. [145].

Li transport is not only important in electrode materials, but also in the electrolyte and in electrode-electrolyte interphases. Solid-state batteries (SSB) are a class of prospective high-energy-density LIBs in which the conventional liquid electrolytes are replaced with solid Li ion conductors [217, 218]. The Li transport in such solid electrolytes and across electrode/electrolyte interfaces is crucial for the performance of SSBs.

In recent work, ML potentials have been employed to investigate Li diffusion in crystalline and non-crystalline solid electrolytes, which are otherwise challenging to model with QM methods. Wang et al. used an MTP based ML potential to carry out long MD simulations of Li diffusion in eight different prospective coating materials for electrodes in SSB [153]. The authors made use of an on-the-fly learning approach to accelerate QM based ab initio MD simulations and to enable simulating long time scales of up to 2 nanoseconds. Fig. 15 shows an Arrhenius plot with a comparison of the Li diffusivities from AIMD, which have large uncertainties due to the limited sampling, with the more accurately determined diffusivities from ML potential simulations. The study also identified cases for which the Arrhenius law did not hold up to the temperatures accessible by DFT, demonstrating that long MD simulations at low temperatures are needed to observe the relevant diffusion behavior.

Amorphous lithium phosphate (${\hbox {Li}_{3}\hbox {PO}_{4}}$) is a Li ion conductor with potential applications as solid electrolyte in all-solid batteries [219], that has attracted much interest because of its chemical and electrochemical stability. Li, Watanabe et al. employed ANN potential based MD simulations to investigate Li transport in amorphous ${\hbox {Li}_{3}\hbox {PO}_{4}}$ [149], considering also large structure models with up to $\sim$1,000 atoms and Li off-stoichiometries. The activation energies for Li diffusion were estimated to be $\sim$0.55 eV, in good agreement with experiment. Nitrogen-doped amorphous ${\hbox {Li}_{3}\hbox {PO}_{4}}$ (LiPON) exhibits better Li conductivity than pristine ${\hbox {Li}_{3}\hbox {PO}_{4}}$ and was investigated using a combination of ANN potentials and DFT calculations by Lacivita et al. [214]. The authors employed an ANN-potential augmented sampling approach with an evolutionary algorithm to determine low-energy amorphous LiPON structure models, which were subsequently recomputed with DFT to ensure accuracy. Fig. 15b shows a representative structure model and the DFT formation energies for different amounts of N doping. The study concluded that amorphous LiPON is generally metastable and decomposition into ${\hbox {Li}_{2}\hbox {O}}$, ${\hbox {Li}_{3}\hbox {PO}_{4}}$, and ${\hbox {Li}_{2}\hbox {PO}_{2}\hbox {N}}$ is thermodynamically favored. The comparison of two different reaction pathways for N doping showed, furthermore, that N substitution with simultaneous Li removal is energetically most likely.

Another example of transport simulations using ANN potentials is the work by Li et al.  who modeled Cu diffusion in amorphous ${\hbox {Ta}_{2}\hbox {O}_{5}}$ [220]. In this study, the ANN potential was trained only on the energy differences caused by Cu intercalation, thereby reducing the complexity of the potential energy surface [220].

While the direct modeling of ionic diffusion with ML potentials is a powerful approach to investigate transport mechanisms, the computational screening for novel ionic conductors does not necessarily require the full complexity of atomistic diffusion simulations. We note, therefore, that ML has also been proposed for the discovery of solid-state Li ion conductors without explicit simulation. Two examples of such materials discovery applications are a study based on unsupervised learning by Zhang et al. [221] and a transfer-learning approach applied to billions of candidate materials by Cubuk et al. [222].

## Remaining challenges and outlook

There are still several remaining challenges in the construction and applications of QM-based ML approaches that we expect to be addressed in future developments. Specifically, the construction of ML models *(1)* still relies on manual validation to ensure reliability and transferability, and *(2)* requires large data sets from QM calculations that may incur computational overheads.

The construction of ML models such as ML potentials requires large reference data sets that come with a computational overhead. It is therefore important to decide first whether a specific research question can be directly addressed with QM based calculations. An ML model is only cost-effective if that is not possible, or if the cost of the QM calculations would exceed the cost of producing the reference data and training an ML model. Note that some applications require length or time scales that cannot directly be accessed with QM methods but may be investigated using more efficient ML models because of the linear scaling of their computational cost (Fig. 8), such as the modeling of nanoparticles reviewed in Sects. “Spectroscopic techniques for structure characterization” and  “Properties of catalyst materials”.

It is important to keep in mind that ML models are only as good as the reference data that they were trained on, and, for example, ML potentials trained on DFT data will generally exhibit the same inaccuracies as the original DFT method.

Careful validation is needed, since the flexibility of the employed ML approaches leads to poor performance in describing data that lies outside the trained range, which can result in stability issues when new regions or conditions are explored. Possible solutions are the inclusion of additional local information (such as forces, curvatures, electronegativity, etc.) and physical constraints in the training process or the use of automated frameworks that generate only relevant structures that improve the description of configurations at the boundary of the training region.

Another challenge is data scarcity due to the high computational cost of the QM reference calculations, especially in the case of unstructured systems that cannot be easily described by simplified models, such as proteins in solution. A partial solution already used in several of the discussed applications is to employ fragmentation approaches in which large molecules are divided into smaller fragments for which QM calculations are more feasible [168, 175, 223]. Transfer learning techniques can also be used to reduce the number of reference calculations, for example by training a model to a more efficient lower-level method first before re-training on a smaller data set obtained from a more expensive higher-level method [224]. Other possibilities are the use of multi-task techniques, in which generalization performance is improved by simultaneously training on multiple related tasks, which could be for example applied to the spectroscopy models summarized in Sect. “Spectroscopic techniques for structure characterization”.

Finally, one could also imagine to completely circumvent the need to run converged MD simulations and employ hybrid approaches in which an ML model learns to predict a converged property from a small number of MD snapshots [225].

Despite the remaining challenges, the impressive applications reviewed in the previous chapter demonstrate that QM-based ML approaches can now be applied to the complex systems required to simulate realistic processes of industrial relevance. It is now possible to obtain a diverse set of properties such as solvation free energies, vibrational spectra, phase diagrams, and transport coefficients with increased efficiency and accuracy, approaching the top left corner in Fig. 1. The rapidly growing number of ML simulations and models, most of which have just been published in the last few years, is a consequence of significant methodological advances, including transferable descriptors and automated training procedures, and the availability of open-source tools. Additionally, community efforts have given rise to public repositories that facilitate the exchange of ML models and data sets. We compiled an extensible list of public tools, data sources, and repositories at https://github.com/atomisticnet/tools-and-data. Together, these resources offer exciting opportunities for knowledge transfer and for exploration of new ML applications in academia and in industry.
